# Glucose-dependent insulinotropic polypeptide receptor signalling in oligodendrocytes increases the weight loss action of GLP-1R agonism

**DOI:** 10.1016/j.cmet.2025.07.009

**Published:** 2025-08-13

**Authors:** Robert Hansford, Sophie Buller, Anthony H Tsang, Simon Benoit, Anna G Roberts, Emmy Erskine, Thomas Brown, Valentina Pirro, Frank Reimann, Norio Harada, Nobuya Inagaki, Ricardo J Samms, Johannes Broichhagen, David J. Hodson, Alice Adriaenssens, Soyoung Park, Clemence Blouet

**Affiliations:** 1 Wellcome-MRC Institute of Metabolic Science, Addenbrookes Hospital, Cambridge CB20QQ; 2 Diabetes, Obesity and Complications, Lilly Research Laboratories, Eli Lilly and Company; 3 Department of Diabetes, Endocrinology and Nutrition, Kyoto University, Kyoto, Japan; 4 Department of Neuroscience, Physiology, and Pharmacology, University College London, London, United Kingdom; 5 Leibniz-Forschungsinstitut für Molekulare Pharmakologie (FMP), 13125 Berlin, Germany; 6 Oxford Centre for Diabetes, Endocrinology and Metabolism (OCDEM), NIHR Oxford Biomedical Research Centre, Churchill Hospital, Radcliffe Department of Medicine, University of Oxford, Oxford, UK

**Keywords:** Glucose-dependent insulinotropic peptide, oligodendrocytes, hypothalamus, median eminence, obesity, weight loss, incretin

## Abstract

The next-generation of obesity medicines harness the activity of the glucose-dependent insulinotropic polypeptide and glucagon-like peptide 1 receptors (GIPR, GLP-1R), but their mechanism of action remains unclear. Here, we report that the GIPR is enriched in oligodendrocytes and GIPR signalling bidirectionally regulates oligodendrogenesis. In mice with adult-onset deletion of GIPR in oligodendrocytes, GIPR agonism fails to enhance the weight-loss effects of GLP-1R agonism. Mechanistically, GIPR agonism increases brain access of GLP-1R agonists, and GIPR signalling in oligodendrocytes is required for this effect. In addition, we show that vasopressin neurons of the paraventricular hypothalamus are necessary for the weight-loss response to GLP-1R activation, targeted by peripherally-administered GLP-1R agonists via their axonal compartment, and this access is increased by activation of the GIPR in oligodendrocytes. Collectively, our findings identify a novel mechanism by which incretin therapies may function to promote synergistic weight loss in the management of excess adiposity.

## Introduction

Glucose-dependent insulinotropic polypeptide (GIP) is a gut hormone released in response to the ingestion of glucose and fat. In addition to its well described action on the incretin axis^1^, activation of the GIPR exerts pleiotropic metabolic benefits, which include improved lipid handling and storage ^2^, appetite suppression and weight loss through its action in the brain ^3^, in particular when combined with long-acting GLP1R agonists (LAGLP1RA) ^4-6^. Multi-receptor agonists engaging the GIPR and GLP1R represent a significant advance in the pharmacological management of excess adiposity, producing substantial weight loss (>20%) in people with obesity, yet how GIPR activation signals in the brain to enhance the efficacy of GLP1R agonism remains unclear.

The GIPR is expressed in discrete brain nuclei important to the regulation of energy homeostasis, including the mediobasal hypothalamus (MBH), which includes the ME and arcuate nucleus of the hypothalamus (ARH), and the area postrema in the hindbrain ^7^. Following peripheral administration, fluorescently-labelled GIPR agonists are detected almost exclusively in the ME, area postrema and choroid plexus, ranking the ME and area postrema as top candidate brain sites mediating the central action of GIPR agonism ^8^. Intriguingly, recent single-cell transcriptomics analysis revealed that the *Gipr* is enriched in the oligodendrocyte populations of the murine MBH and hindbrain ^9 10 7,11,12^, and human hypothalamus ^13^, but how GIPR signalling affects oligodendrocyte biology, and whether this might contribute to the metabolic benefits associated with GIPR activation is unknown. While the area postrema is enriched in GIPR-expressing neurons, and GIPR agonism is believed to mediate its anti-aversive activity via this area of the brain ^8 14 15 16^, the ME mostly contains non-neuronal cells, including a population of highly plastic oligodendrocytes which constitutively turnover in adulthood and are responsive to acute and chronic nutritional and metabolic signals ^11,17^.

Here we used a combination of approaches to interrogate the role of the GIPR in adult oligodendrocyte biology and examine the contribution of GIPR signalling in oligodendrocytes to the weight loss efficacy of GIPR and GLP1R agonism in diet-induced obese mice. We provide evidence that the GIPR is expressed in myelinating oligodendrocytes and enriched in oligodendrocytes of the ME. Oligodendrocyte-specific deletion of the GIPR in adulthood reduces adult oligodendrogenesis and oligodendrocyte survival in the ME, while treatment with GIPR agonists promotes ME oligodendrogenesis, supporting the functional relevance of GIPR activation in the regulation of oligodendrocyte and myelin plasticity in adult mice. We find that expression of the GIPR in oligodendrocytes is required for GIPR agonism to enhance the weight-loss and appetite-suppressive effects of GLP1R agonism. Mechanistically, GIPR agonism increases access of GLP1R pharmacotherapies to the ME and ARH by increasing local VEGF-A expression and vessel fenestration, and GIPR expression in oligodendrocyte is required for GIPR agonism to enhance access of GLP1R agonists to these regions. Collectively, our findings identify a novel mechanism by which GIPR/GLP-1R-based therapies may function via the GIPR to augment GLP-1R activation in the brain and promote synergistic weight loss, when compared to selective GLP-1R activation.

## Results

### The *Gipr* is enriched in median eminence oligodendrocytes

**Figure 1:**
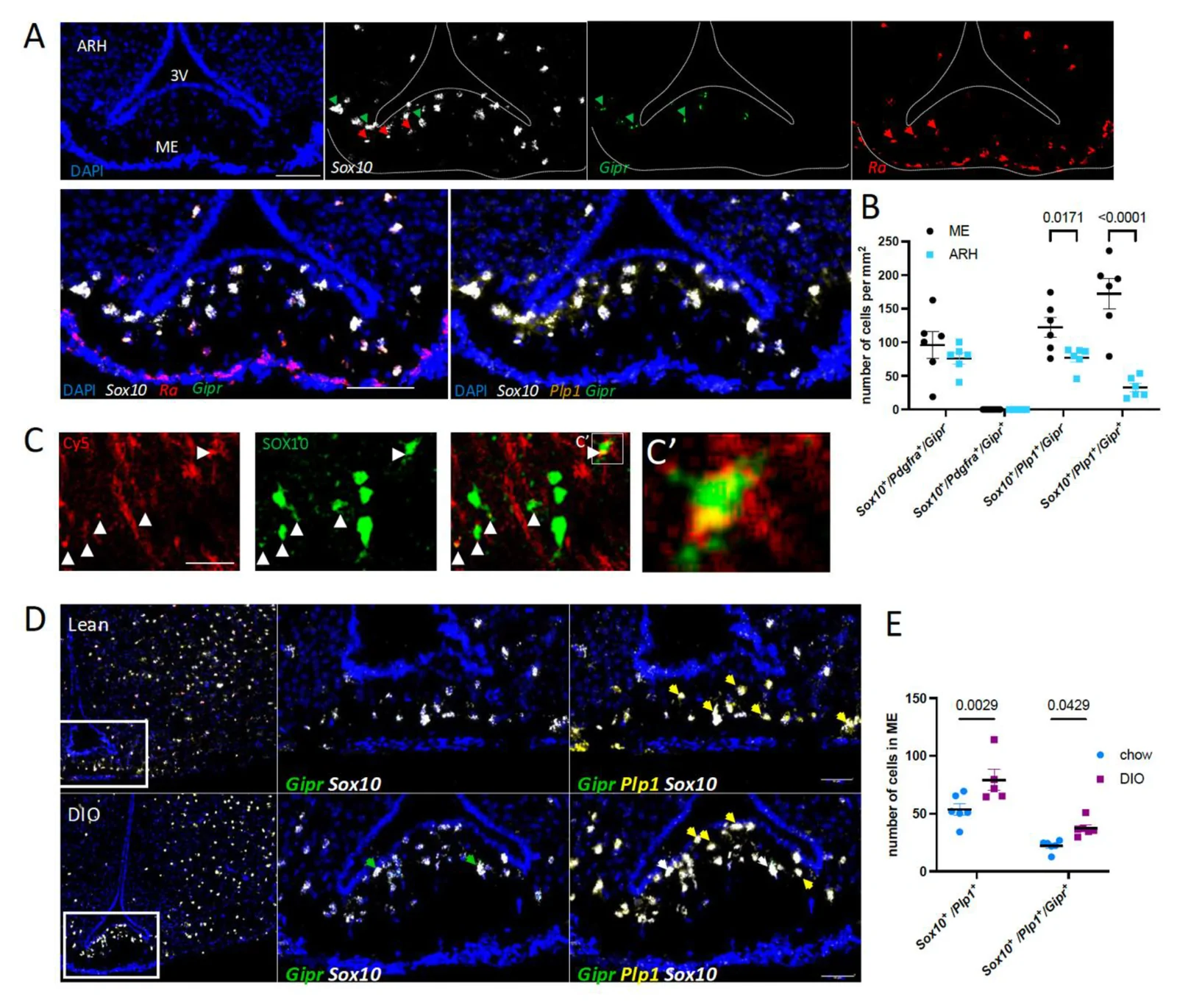
The *Gipr* is enriched in murine and human ME mature oligodendrocytes. Representative images **(A)** and quantifications **(B)** of FISH detection of *Gipr* (green) in oligodendrocyte lineage cells using *Sox10* to label the entire lineage (white), *Pdgfra* (Ra, red) to label OPCs (Sox10^+^/Ra^+^, red arrowheads), and *Plp1* (yellow) to label oligodendrocytes in the mouse median eminence. *Gipr* expression oligodendrocytes (*Gipr*^+^/Sox10^+^) are indicated by the green arrowheads. Each datapoint is the average of 3 sections per animals. Scale bar is 50um. **(c)** Immunofluorescent detection of the labelled GIPR agonist (GIPRA^Cy5^, red) and colocalization with oligodendrocyte marker *Sox10* (green) in the mouse ME following intravenous administration. Scale bar is 20µm. Representative images (**D)** and quantification (**E)** of FISH detection of *Gipr* (green, green arrowheads) in oligodendrocyte lineage cells labelled with *Sox10* (white) and *Plp1* (yellow, yellow arrowheads) to label oligodendrocytes in the mouse ME from mice fed a standard chow (lean) of 60% HF-diet (DIO) for 8 weeks from P60. Each datapoint is the average of 3 sections per animals. Green arrowheads: *Gipr^+^ /Sox10^+^* cells; Yellow arrowheads: *Gipr^+^/Sox10^+^/Plp1^+^* cells Scale bar is 50µm. Data are presented as mean ± sem and analysed by Student’s t-test. See also Figure S1 and Table S1-S2.

We first conducted a comprehensive evaluation of *Gipr* expression in oligodendrocyte lineage cells of the MBH, dorso-vagal complex (DVC) and main white matter tracts (corpus callosum - CC, optic nerve, amygdala, striatum, substantia nigra, VTA, dorsal horn) in adult male mice, using multiplexed fluorescent *in situ* hybridization (FISH, RNAscope technology) against the *Gipr, Sox10, Pdgfra* and *Plp1*. Consistent with published transcriptomics datasets ^7,9,10,11^, we found very rare expression of the *Gipr* in oligodendrocyte progenitor cells (OPCs, *Sox10^+^/Pdgfra^+^*) across all examined regions (Fig. 1A-1B, S1A). In contrast, the *Gipr* was present in oligodendrocytes (*Sox10^+^/Plp1^+^*). The proportion of *Gipr*^+^ oligodendrocytes varied between regions and was significantly higher in the median eminence (ME) than the adjacent hypothalamic parenchyma (arcuate nucleus of the hypothalamus, ARH) or all main white matter tracts (Fig. S1B). To gain insights into the molecular identity of the subset of ME oligodendrocytes expressing *Gipr*, we used our published single-cell transcriptomic dataset of the mouse murine ME ^11^ and analysed genes differentially expressed between *Gipr*^-^ and *Gipr*^+^ oligodendrocytes (Table S1). Top differentially expressed genes included *Klk6* and *Phlda1,* enriched in mature oligodendrocytes ^18^, *Marcks*, a regulator of oligodendrocyte maturation ^19^ and interferon response gene *Ifi27l2a*, collectively suggesting an enrichment of the *Gipr* in mature myelinating oligodendrocytes. Pathway analysis of genes enriched in *Gipr*^+^ oligodendrocytes revealed an enrichment in genes involved in adipogenesis and estrogen receptor signalling (Table S2). Next, we used a labelled GIPR agonist (GIPRA^Cy5^) to examine the distribution of the GIPRA in the ME. Following intravenous administration, the GIPRA^Cy5^ was found in close proximity to SOX10^+^ oligodendrocyte lineage cells in the ME. This indicates that peripherally dosed GIPR-based therapeutics are capable of targeting oligodendrocytes in the MBH (Fig. 1C).

### *Gipr* expression is upregulated in ME oligodendrocytes of diet-induced obese mice

In lean healthy mice, the oligodendrocyte population in the ME turns over rapidly, with approximatively 50% being replaced within 6 weeks ^17^, while high-fat feeding blunts ME oligodendrocyte turnover, leading to an accumulation of mature oligodendrocytes in the ME ^17^. Since the *Gipr* is enriched in mature oligodendrocytes (Table S1), we evaluated whether diet-induced obesity (DIO) leads to an increase in the number of *Gipr-*expressing oligodendrocytes in the ME. Consistent with our previous work, we found a 50% increase in ME oligodendrocyte density in mice maintained on a 60% high-fat (HF) diet when compared to age-matched chow-fed controls (average body weight 28.6 ± 1.6 vs 48.8 ± 2.2g in chow and HF-fed mice, respectively (Fig. 1D). This change was specific to the ME and not observed in any other inspected regions (ARH, NTS, AP, DMX, CC, Optic nerve, amygdala, striatum, substantia nigra, VTA, dorsal horn – not shown). As predicted, DIO increased the number of oligodendrocytes expressing the *Gipr* in the ME (Fig.1E), but not in other regions (Fig. S1C-S1D).

### GIPR signalling in oligodendrocytes regulates adult ME oligodendrogenesis in HF-fed mice

To characterise the role of GIPR signalling in oligodendrocytes during the development of DIO, we generated mice with adult-onset oligodendrocyte-specific *Gipr* deletion using *Plp1-CreER^T2^* mice crossed with *Gipr^fl/fl^* mice ^20^ (OL^GIPR-/-^) and wild-type littermates (OL^GIPR+/+^). At postnatal day 60 (P60), mice received 8 daily ip doses of tamoxifen (Tm, 80 mg/kg) to induce Cre expression in oligodendrocytes and were switched to a 60% HF-diet 2 weeks later. MBH micro-punches were collected at 3 and 15 weeks post Tm treatment to measure MBH *Gipr* expression. HF-feeding produced a significant increase in MBH *Gipr* expression in OL^GIPR+/+^ mice, consistent with RNAscope analysis (Fig. 1E), which did not occur in OL^GIPR-/-^ mice, resulting in a 40% reduction in MBH *Gipr* expression in OL^GIPR-/-^ compared to OL^GIPR+/+^mice (Fig. S2A). FISH detection of *Gipr* in the ME of HF-fed OL^GIPR-/-^ mice confirmed OL-specific deletion under these conditions (Fig. S2C)

**Figure 2:**
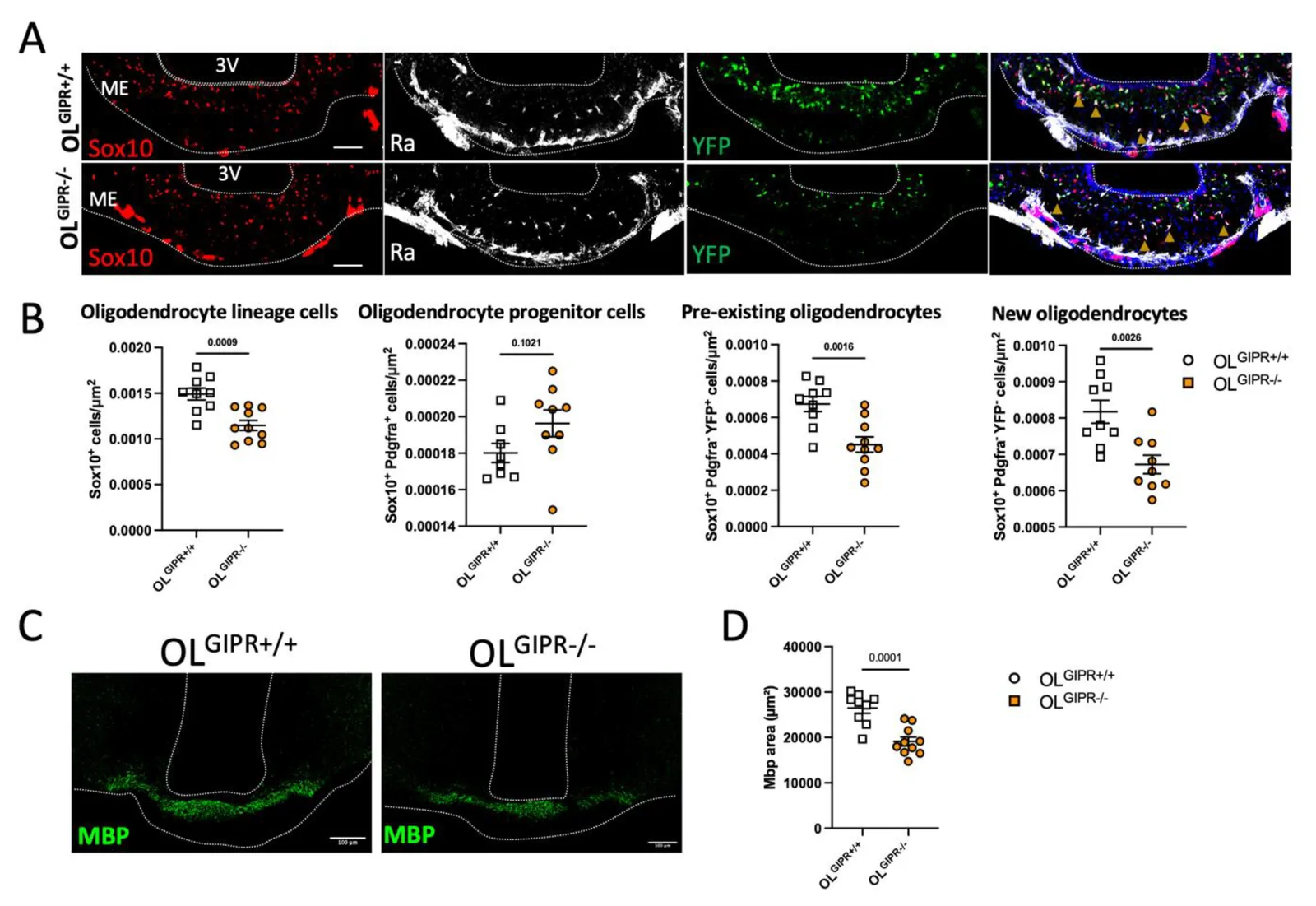
GIPR signalling in oligodendrocytes regulates ME oligodendrogenesis in HF-fed mice. Representative images **(A)** and quantification **(B)** of oligodendrocyte lineage cells labelled with YFP at P60 for fate-mapping purposes and detected using multiplexed immunofluorescent detection of SOX10 (red), PDGF receptor agonist (Ra, white), and YFP (green) in the ME of HF-fed OL^GIPR +/+^ and OL^GIPR -/-^ mice. Scale bar is 50µm. Yellow arrow heads show OPCs, coexpressing *Sox10* and *Pdgfra*. Representative images **(C)** and quantification **(c)** of MBP (green) in the ME of HF-fed OL^GIPR +/+^ and OL^GIPR -/-^ mice. Scale bar is 100µm. Data are presented as mean ± sem and analysed by Student’s t-test. See also Figure S2.

OL^GIPR-/-^ and OL^GIPR+/+^ were further crossed with a GFP reporter line (*R26-Yfp*) to label the oligodendrocyte population present at the time of Tm administration at P60 and measure the effect of *Gipr* KO on ME oligodendrocyte lineage cell plasticity and longevity in mice maintained on a 60% HF diet (Fig. S2B). In OL^GIPR-/-^mice 12 weeks after Tm administration, there was a significant reduction in the density of oligodendrocyte lineage cells (SOX10^+^) in the ME, with no change in the density of OPCs (SOX10^+^/PDGFRa^+^), suggesting a reduction in the density of oligodendrocytes (Fig. 2A, 2B). Further, we found a significant reduction in the density of both oligodendrocytes present in the ME at the time of Tm administration (pre-existing oligodendrocytes, SOX10^+^/PDGFRa^-^/YFP^+^, Fig 2A, 2B) and oligodendrocytes generated from OPC differentiation after Tm administration (new oligodendrocytes, SOX10^+^/PDGFRa^-^/YFP^-^, Fig 2A, 2B), suggesting that GIPR activation in oligodendrocytes promotes oligodendrocyte longevity and adult oligodendrogenesis in the ME of HF-fed mice. Of note, because the *Plp1-CreER^T2^* allele yields to 70% recombination^17^, the YFP^-^ population also reflects oligodendrocytes which did not undergo recombination. Consistent with a role for ME oligodendrogenesis in the maintenance of ME myelin in adulthood ^17^, reduced oligodendrogenesis and longevity of ME oligodendrocytes in OL^GIPR -/-^ mice was associated with reduced expression of myelin basic protein (MBP) in the ME, a standard marker for visualising myelin sheaths ^21^, suggesting reduced myelination (Fig. 2C, 2D). In contrast, density of oligodendrocyte lineage cells was similar between groups in the CC (Fig. S2D, S2E), indicating that responsiveness to peripheral GIPR activation is a feature of ME oligodendrocytes.

### GIPR signalling in oligodendrocytes controls energy expenditure and insulin sensitivity in HF-fed mice.

**Figure 3:**
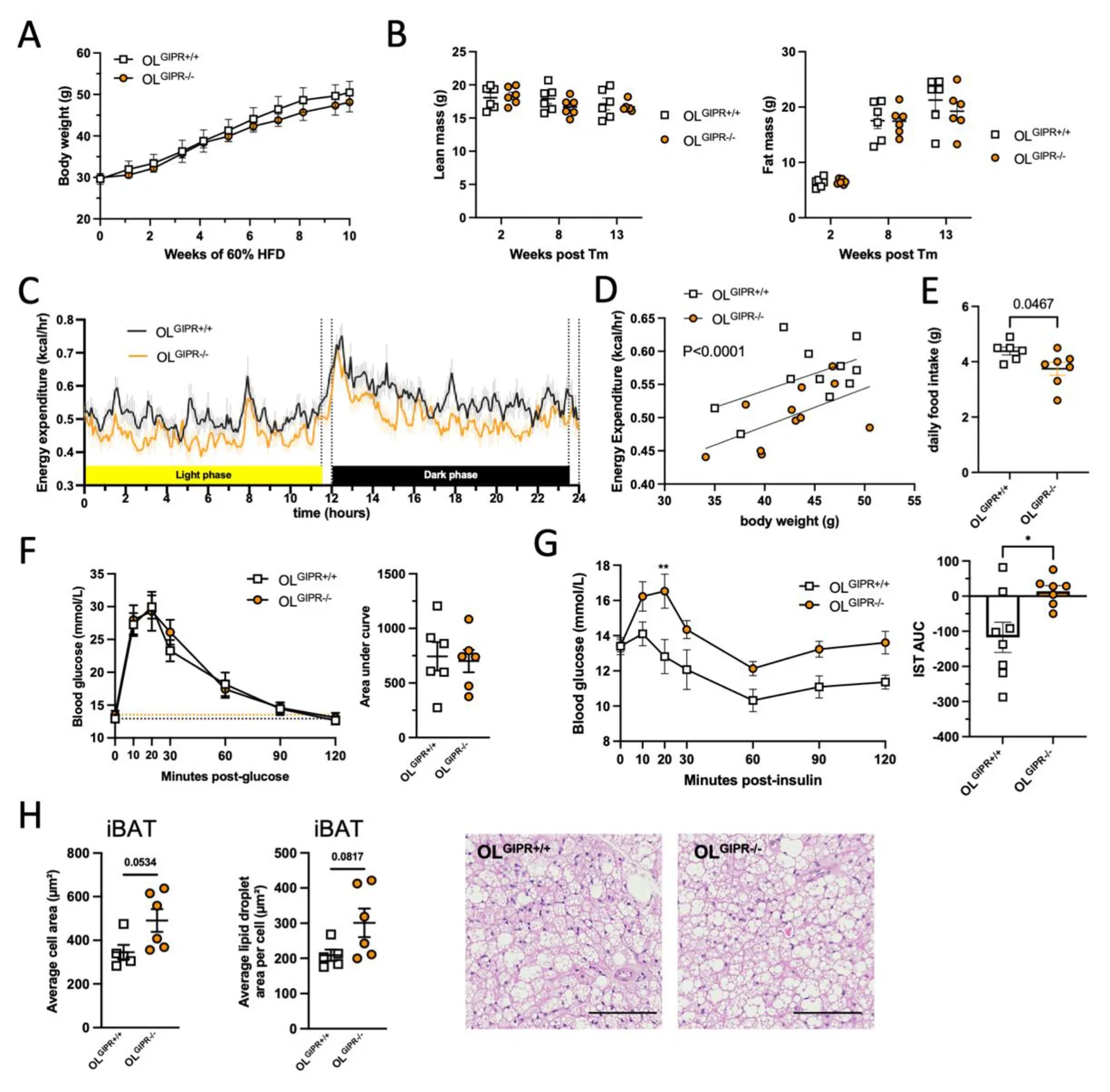
GIPR signalling in oligodendrocytes regulates energy expenditure and insulin sensitivity in HF-fed mice. Body weight gain (**A**), body composition (**B**), energy expenditure (**C**), correlation between energy expenditure and body weight (**D**), energy intake (**E**), blood glucose excursion during an oral glucose tolerance test (**F**) or ip insulin tolerance test (**G**) and cellularity analysis of iBAT adipocytes (**H**) in the metabolic phenotyping cohort of OL^GIPR -/-^ and OL^GIPR +/+^ mice maintained on 60% HF diet. Scale bar is 50um. Data are presented as mean ± sem and analysed by 2-way ANOVA (A-G) or student’s t-test (H). See also Figure S3, Table S3.

We next examined the metabolic phenotype of OL^GIPR -/-^ mice maintained on a 60% HF-diet. Body weight gain and body composition did not differ between OL^GIPR -/-^ mice and WT littermates (Fig 3A, 3B). However, deletion of the GIPR in oligodendrocytes significantly reduced energy expenditure and energy intake (Fig. 3C-3E). Substrate utilisation (RER, respiratory exchange ratio) and locomotor activity did not differ between groups (Fig. S3A-B). OL^GIPR -/-^ mice maintained glycaemic control similar to OL^GIPR +/+^ mice in response to an oral glucose challenge (Fig. 3F) but had impaired insulin tolerance following an intraperitoneal dose of insulin bolus (Fig. 3G), suggesting impaired insulin sensitivity. No significant changes were found in liver and epididymal white adipose tissue (eWAT) cellularity (Fig. S3C-3D), but there was a trend towards larger adipocytes in the interscapular brown adipose tissue (iBAT) and increased lipid droplet area in the iBAT of OL^GIPR-/-^ mice (Fig. 3H). Metabolomics analysis of plasma and tissue samples from OL^GIPR-/-^ and OL^GIPR+/+^ mice using MANOVA indicated clear group segregation of the overall metabolite profile (Fig. S3E, Suppl Table 3). Univariate statistical analysis highlighted changes in concentrations of circulating and tissue amino acid and related metabolites concentrations, in particular metabolites involved in branched chain amino acid (BCAA) metabolism such as isovalerylcarnitine, beta-hydroxyisovaleric acid, N-acetylleucine, 3-methyl-2-oxovaleric acid, 2-hydroxyisocaproic acid or 3-hydroxyisobutyric acid, all of which decreased in the adipose tissue of OL^GIPR-/-^ mice (p<0.05 in eWAT, p<0.1 in iBAT) (Fig. S3F, Suppl Table 3). Consistently, correlation network analysis ^22^ highlighted significant changes in BCAA metabolism in OL^GIPR-/-^ compared to WT mice (Suppl Table 3). Thus, despite the lack of a net effect on body weight, these data support a role for endogenous GIPR signalling in oligodendrocytes in the regulation of whole-body energy homeostasis, glycaemic control and BCAA utilisation in HF-fed mice.

### GIPR agonism promotes ME oligodendrogenesis and oligodendrocyte turnover

GIPR agonism has emerged as an effective therapeutic strategy in the metabolic field, through its ability to potentiate the appetite- and weight-lowering action of GLP1R agonism. However, the underpinning mechanisms are incompletely understood. To determine whether GIPR agonism mediates its therapeutic activity via ME oligodendrocytes, we examined the consequences of treatment with a long-acting GIPR agonist (LAGIPRA, 300nmol/kg ^23^) in *Opalin-iCreER^T2^:tdTom* male mice. In these mice, Tm administration at P60 induces tdTom expression specifically in mature oligodendrocytes, allowing us to quantify oligodendrocyte survival and new oligodendrocyte production ^24^. Mice were dosed with the LAGIPRA for 2 weeks shortly after Tm administration on chow maintenance, following 8-weeks on a 60% HF diet to induce DIO (Fig. S4A, S4B).

In chow-fed lean mice, GIPR agonism did not significantly impact weight gain (Fig. S4C) but significantly increased the number of oligodendrocyte lineage cells in the ME, with both an increase in OPC and oligodendrocyte density over 2 weeks (Fig. S4E, S4F). We measured both an increase in the number of oligodendrocytes labelled with td-tom, indicating increased survival of ME oligodendrocytes, and an increase in TdTom^-^ oligodendrocytes, indicating increased oligodendrogenesis (Fig. S4E, S4F). Consistent with these changes, ME MBP immunolabelling increased in mice dosed with the LAGIPRA (Fig. S4G, S4H). Thus, GIPR agonism increases oligodendrogenesis and oligodendrocyte survival in the ME in lean chow-fed mice (Fig. S4K).

**Figure 4:**
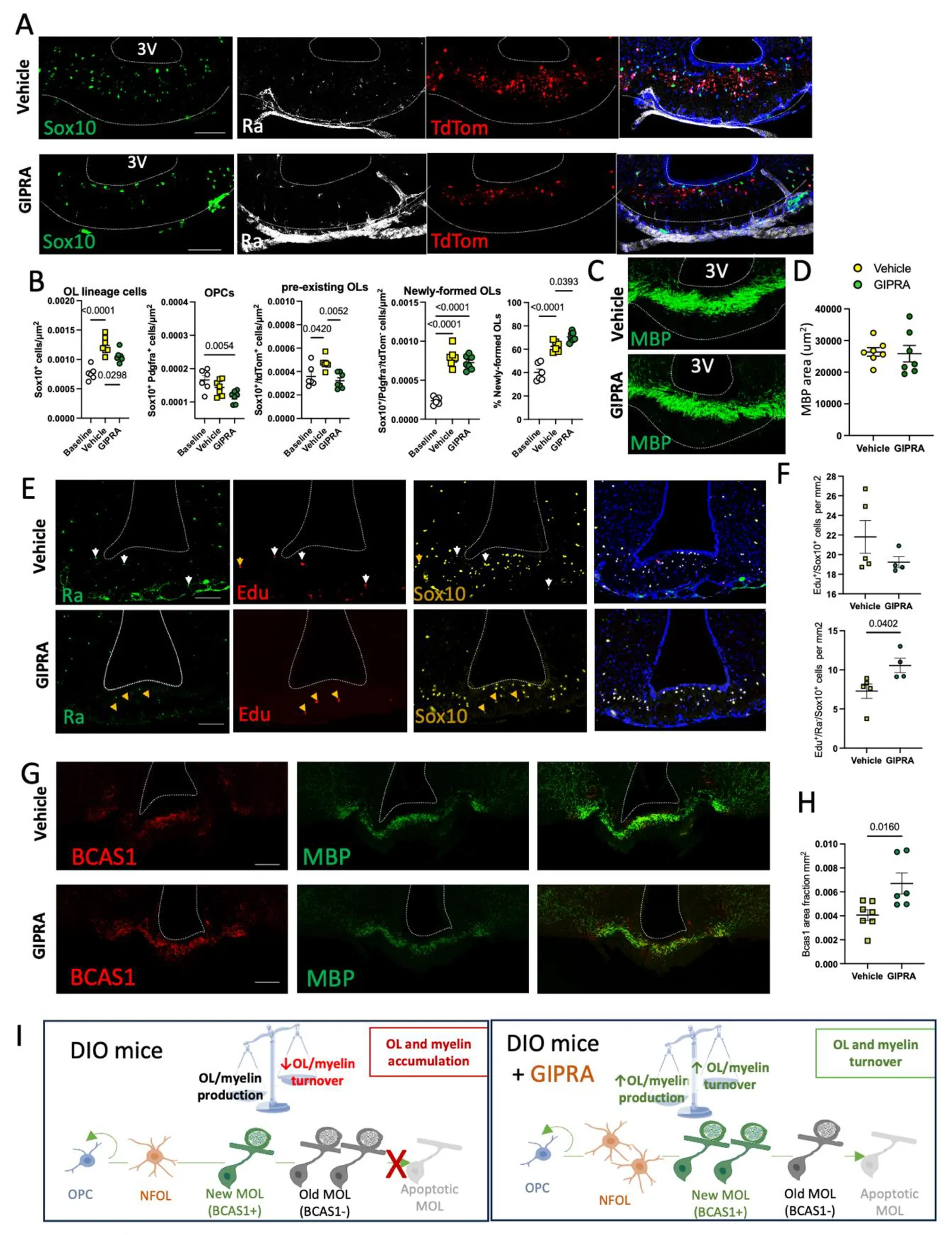
GIPR agonism increases ME oligodendrogenesis and oligodendrocyte turnover. Representative images (**A, C**) and quantification (**B, D**) of the immunoreactivity against SOX10 (green), PDGF receptor agonist (Ra, white), TdTom (red) and MBP (green) in the ME of HF-fed *Opalin-iCreER^T2^:TdTom* mice following Tm administration at P60 and 2 weeks of subcutaneous daily dosing with a long-acting GIPR agonist or vehicle. Scale bar is 100um. Representative images (**E**) and quantification (**F**) of the immunoreactivity against SOX10 (yellow), PDGF receptor agonist (Ra, green) and Edu (red) in the ME of HF-fed mice following 2 weeks of subcutaneous daily dosing with a long-acting GIPR agonist or vehicle, and 4 doses of intraperitoneal EdU over 24hr just before terminal perfusion. Scale bar is 100um. Representative images (**G**) and quantification (**H**) of the immunoreactivity against BCAS1 (red) in the ME of HF-fed mice following 2 weeks of subcutaneous daily dosing with a long-acting GIPR agonist or vehicle. Scale bar is 100um. (**I**) Summary schematic of the effect of GIPR agonism on the ME oligodendrocyte lineage in DIO mice. Data are presented as mean ± sem and analysed with Student’s t-test. See also Figure S4.

In DIO mice, chronic treatment with the LAGIPRA produced a modest but significant decrease in body weight (Fig. S4D). As previously reported, DIO significantly increased ME oligodendrocyte density ^17^, but this response was blunted in the LAGIPRA treated mice (Fig. 4A, 4B). Since oligodendrocyte density results from both oligodendrogenesis and oligodendrocyte turnover in the ME ^17^, we used complementary approaches to quantify both processes. The proportion of newly-formed oligodendrocytes was increased following LAGIPRA treatment, suggesting increased oligodendrogenesis (Fig. 4A, 4B). This was accompanied by a decrease in OPC density, and an increase in the density of Edu^+^/Pdgfra^-^/Sox10^+^ cells following an 24h EdU pulse at the end of the 2-weeks treatment, suggesting rapid differentiation of recently divided OPCs (Fig. 4E, 4F). In addition, 2 weeks of treatment with the LAGIPRA increased the number of oligodendrocyte lineage cells expressing *Bmp4*, a specific marker for newly-formed oligodendrocytes ^25^, specifically in the ME, further indicating that GIPR agonism in DIO mice increases oligodendrogenesis (Fig. S4I, S4J). We also observed an increase in the density of BCAS1, a specific marker of early-myelinating oligodendrocytes^26^ under these conditions (Fig. 4G, 4H). Moreover in the fate mapping study, we measured a decrease in the density of tdTom^+^ pre-existing OLs (Fig. 4A, 4B) with GIPR agonism, indicating increased oligodendrocyte turnover. As a result of both an increase in oligodendrogenesis and an increase in oligodendrocyte turnover, MBP density was similar between groups (Fig. 4C, 4D). Thus, GIPR agonism promotes ME oligodendrogenesis in DIO mice and restores oligodendrocyte turnover (Fig. 4I).

### GIPR signalling in oligodendrocytes is required for GIPR agonism to enhance the weight loss efficacy of GLP1R agonism in obese mice

Based on our previous finding that adult oligodendrocyte plasticity regulates hypothalamic leptin sensing ^17^, we hypothesised that GIPR agonist-induced changes in ME oligodendrocyte turnover in DIO mice might alter hypothalamic sensing of metabolic signals. If true for hypothalamic sensing of GLP1R agonists, this might represent a novel mechanism by which GIPR activation enhances the weight loss benefits of GLP1R agonism. To explore this possibility, we first examined the role of oligodendrocyte GIPR signalling in the weight-loss action of combined GIPR and GLP1R agonism.

**Figure 5:**
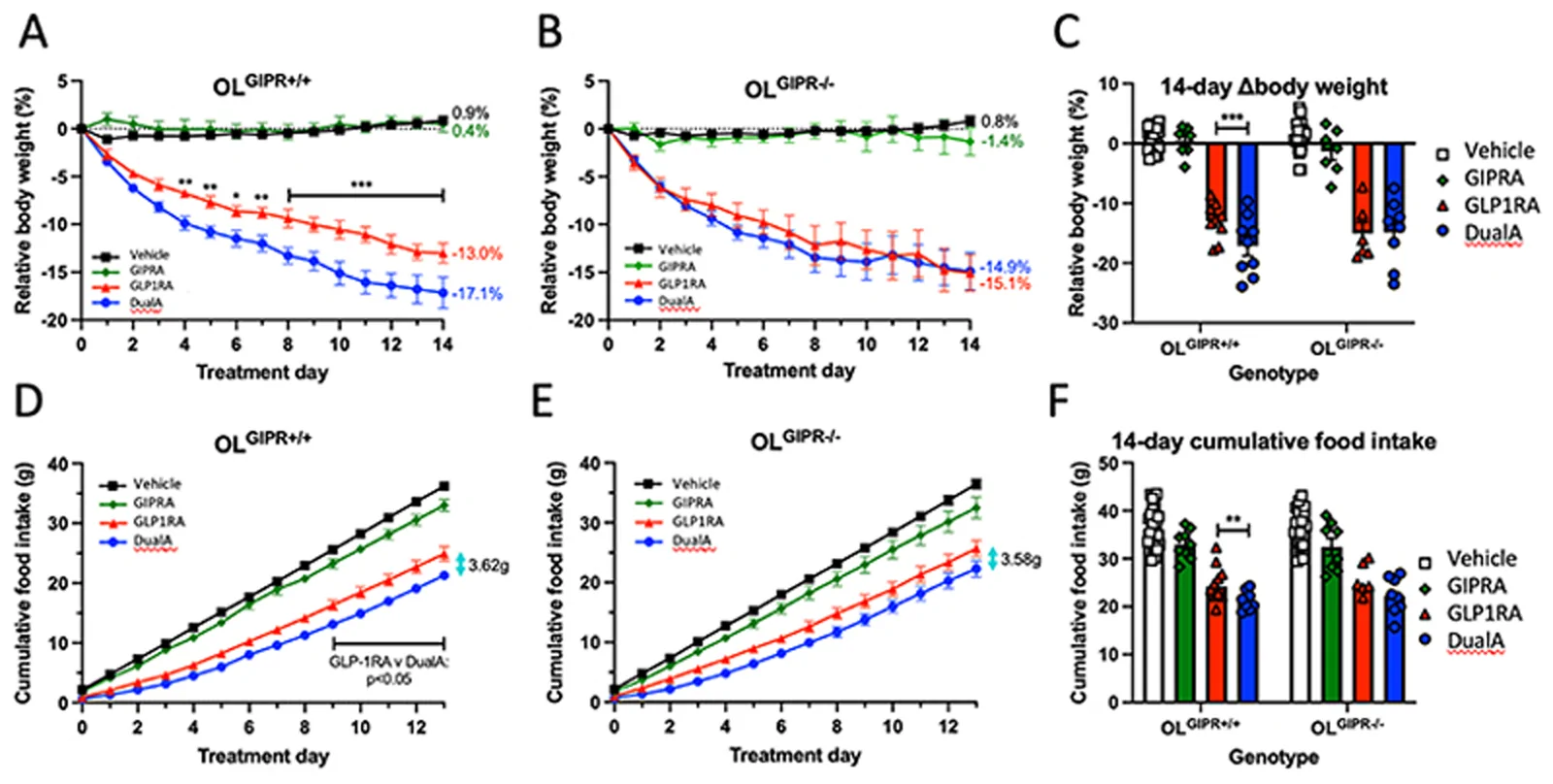
GIPR signalling in oligodendrocytes is required for the beneficial effects of GIPR agonism the anti-obesity actions of GLP1R agonist. Body weight change in OL^GIPR+/+^ (**A, C**) and OL^GIPR -/-^ (**B, C**) mice in response to vehicle, LAGIPRA, LAGLP1RA and combination (DualA) treatments as a proportion of baseline body weight (n=7-9). (**D**) Cumulative food intake in OL^GIPR +/+^ (**D, F**) and OL^GIPR -/-^ (**E, F**) mice in response to vehicle, LAGIPRA, LAGLP1RA and combination (DualA) treatments. Data presented as mean ± sem. GIPR agonism v GLP1R agonism p*<0.05, **<0.01, ***<0.001, ****<0.0001. GIPR agonism v DualA ^$$^<0.01, ^$$$^<0.001, ^$$$$^<0.0001. GLP1R agonism v DualA p^#^<0.05, ^##^<0.01. Data presented as mean ± sem and analysed with a 2-way ANOVA (A,B, D, E) or 1-way ANOVE (C, F). See also Figure S5.

OL^GIPR -/-^ mice and WT littermates were maintained on a 60% HF diet for 10 to 16 weeks to reach an average body weight of 45g. Mice were then dosed subcutaneously with a vehicle for 2 weeks, followed by daily treatment with either a LAGLP1RA alone or in combination (DualA) with the LAGIPRA (Fig. S5A). Again, OL^GIPR -/-^ mice gained a similar amount of body weight as controls during HF-diet maintenance (Fig. S5B). In WT controls, treatment with the LAGLP1RA significantly reduced body weight and food intake, which were amplified by the LAGIPRA (Fig. 5A, 5C, 5D, 5F). In contrast, in OL^GIPR-/-^ mice, the LAGIPRA failed to augment the anorectic or weight loss profile of the LAGLP1RA (Fig. 5B, 5C, 5E, 5F). Thus, our findings suggest that GIPR signalling in oligodendrocytes is required for GIPR agonism to enhance the anti-obesity efficacy of GLP1R agonism in high-fat fed obese mice.

### GIPR signalling in oligodendrocytes increases access of a peripherally dosed LAGLP1R agonist to the MBH

Adult-born oligodendrocytes have been recently shown to modulate hypothalamic neuroendocrine functions in response to hormonal and nutritional signals by regulating the expression of the angiogenic factor VEGF-A, enhancing ME vascular permeability ^27,28^. Based on these findings, we hypothesised that the upregulation of new oligodendrocyte production in response to GIPR agonism might increase vascular permeability at the ME-ARH barrier and enhance the access of circulating molecules to their hypothalamic targets. This could be specifically relevant during co-treatment with GLP1R agonists, which do not cross the blood-brain barrier ^29^. Using brain clearing and light-sheet microscopy to visualise the distribution of a fluorescently labelled short acting GLP1R agonist (SAGLP1RA, Exendin-4) following peripheral administration, we found that peripherally administered GLP1R agonists selectively access the brain through circumventricular organs, with the ME being one of the top brain regions exposed to peripherally administered GLP1R agonists, consistent with the published literature^30,31^ (Fig. S6A, S6B, Suppl. video 1).

**Figure 6:**
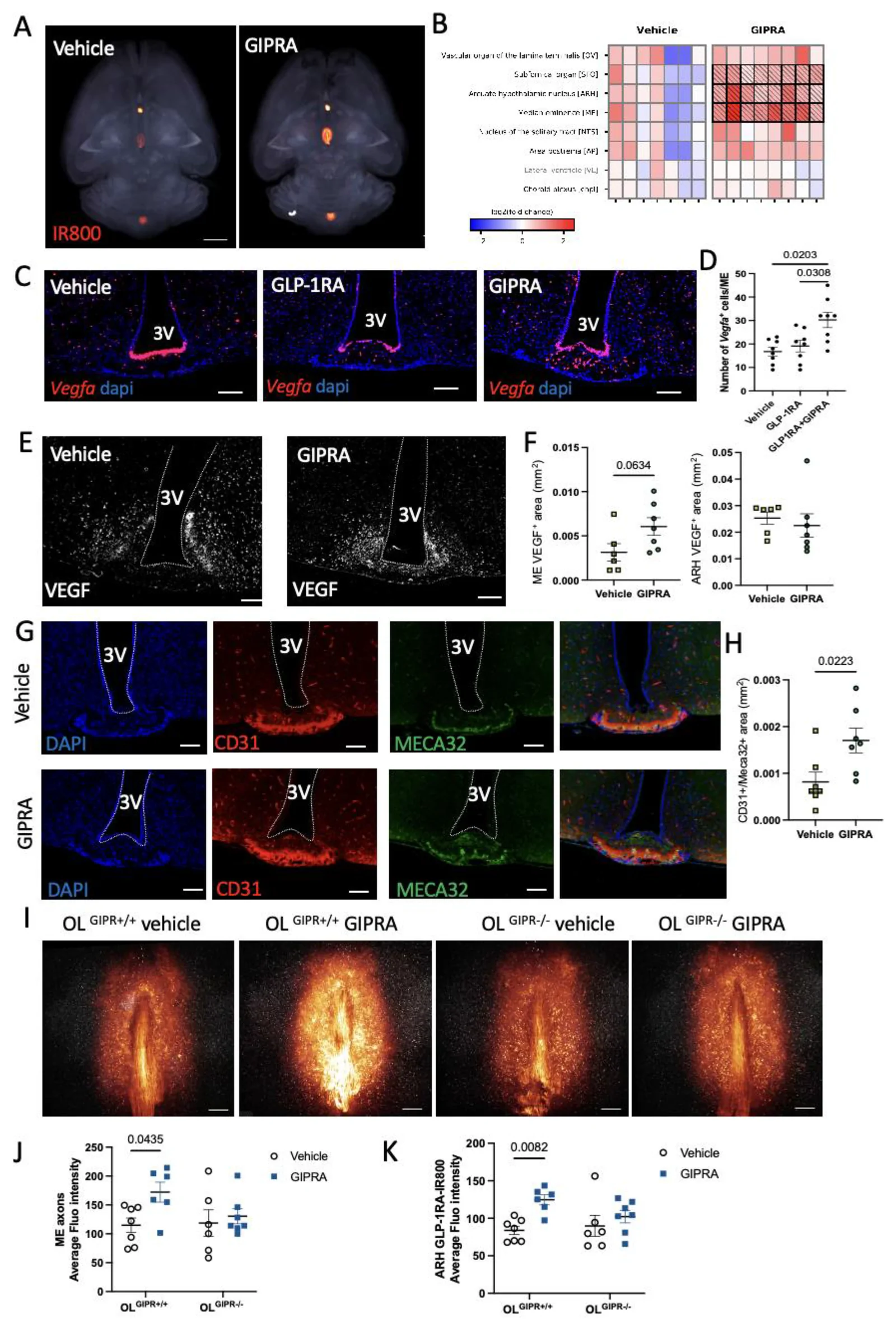
GIPR signalling in oligodendrocytes increases access of a peripherally dosed LAGLP1R agonist to the MBH Representative images (**A**, scale bar is 1mm) and quantification of the fluorescence intensity compared to mean intensity in the vehicle group for each brain region (**B**) of the brain distribution of IR-800 imaged at low resolution in WT mice following a pre-treatment with vehicle or GIPR agonism for 7 days and a terminal bolus with IR800-Ex4. In B, each column represents the fluorescence intensity in 1 individual brain sample. Fluorescence intensity for every animal in the GIPR-treated group is compared to the mean value in the Vehicle treated group and represented as a heatmap. Areas showing statistically significant compound fluorescence intensity changes are highlighted in hashed and bold. Representative images **(C**) and quantification (**D**) of FISH detection of *Vegfa* (red) in the ME of DIO mice treated for 7 days with a GLP1RA alone or in combination with the GIPRA. Representative images (**E**) and quantification (**F**) of VEGF immunodetection in the ME of DIO mice treated for 7 days the GIPRA. Representative images (**G**) and quantification (**H**) of CD31 (red) and MECA32 (green) immunodetection in the ME of DIO mice treated for 7 days with the GIPRA. Scale bar is 100um. Representative images of a ventral view of IR-800 fluorescence in the MBH, imaged with fluorescent light-sheet microscopy at high resolution (**I,** scale bar is 200um), and quantification of IR800 fluorescence in fibers of the ME (J) and adjacent ARH region (K) in HF-fed OLGIPR-/-mice and WT littermates following a pre-treatment with vehicle or GIPR agonism for 7 days and a terminal bolus with IR800-Ex4. Data are presented as mean ± sem (n=6-7) and analysed with a Negative Binomial Generalized Linear Model with Tukey’s test as post-hoc analysis to determine differential signal accumulation between groups with FDR correction (<0.05) (B), Student’s t-test (D, F, H) or 2-way ANOVA (J, K). See also Figure S6.

To test this hypothesis, DIO C57/Bl6J males were treated for 6 days with the LAGIPRA or vehicle and on the day of sacrifice injected with a single dose of SAGLP1RA^IR800^ subcutaneously. Brains were collected and processed for clearing and fluorescent light-sheet microscopy. Treatment with the GIPR agonist significantly increased IR800 fluorescence in the ME, ARH and subfornical area, but not other circumventricular organs (Fig. 6A, 6B,). Thus, GIPR activation increases hypothalamic access of GLP1R agonists. Consistent with a change in ME-ARH vascular permeability, we found an increase in the number of cells expressing *Vegfa* specifically in response to GIPR but not GLP1R agonism (Fig. 6C, 6D). This was associated with an increase in the area immunoreactive for VEGF (Fig. 6E-6F), and an increase in the density of MECA32^+^ fenestrated capillaries under these conditions (Fig. 6G-6H).

We tested the role of GIPR signalling in oligodendrocyte in the increased brain access of GLP1RA following GIPR activation. Tm-dosed OL^GIPR -/-^ mice and WT littermates were maintained on a 60% HF diet for 12 to 16 weeks to reach a minimum body weight of 45g, followed by 7 daily subcutaneous injections with a LAGIPRA or vehicle (Fig. S6C-S6D). On the day following the last dose, all mice received a single intravenous dose of SAGLP1RA^IR800^ and brains were collected and processed for clearing and fluorescent light-sheet microscopy of the MBH at high resolution. In WT mice, 7 days pre-treatment with the LAGIPRA significantly increased IR800 fluorescence intensity in the ME and ARH (Fig. 6I-6K). In contrast, in OL^GIPR -/-^ mice, there was no effect of the LAGIPRA on the uptake of the labelled GLP1RA in the ME and ARH (Fig. 6I-6K). Taken together, our findings indicate that GIPR agonism increases vascular permeability in the ME of DIO mice, facilitating brain access of GLP1R agonists, and that GIPR signalling in ME oligodendrocytes is required to increase the uptake of GLP1RA-based therapeutics in the MBH.

### PVH AVP neurons access peripherally administered GLP1R agonists through their axonal segment in the ME, enriched in GLP1R

**Figure 7:**
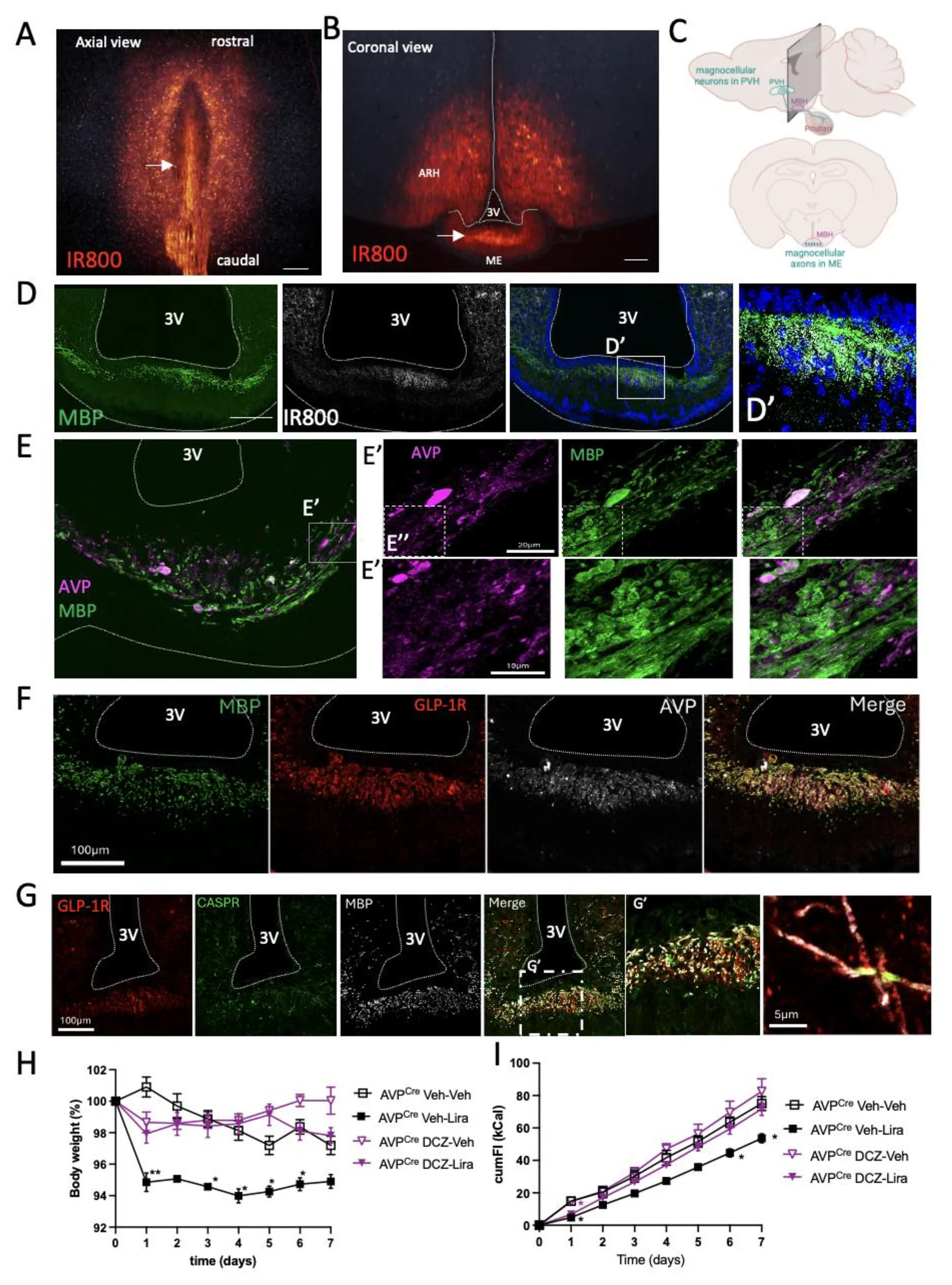
PVH AVP neurons are required for the weight loss response to GLP1R agonism and access peripherally administered GLP1R agonists through their axonal segment in the ME. Ventral view (**A)** and coronal view (**B**) of the MBH of a cleared brain from a mouse injected intravenously with IR-800 GLP1R and imaged at high resolution with light-sheet microscopy, visualized in glow scale and autofluorescence in grey. White arrows indicate myelinated axon bundles. (C) Schematic representation of the orientation of PVH magnocellular axons. (D) Immunodetection of MBP and imaging of IR800 fluorescence in the MBH of a mouse injected intravenously with SAGLP1RA^IR800^. Scale bar is 100 um. Immunodetection of MBP (green) and AVP (magenta) in the mouse MBH imaged with super resolution microscopy at 63X (**E, E”**). (**F**) Immunodetection of GLP1R (red), AVP (white) and MBP (green) imaged with super resolution microscopy at 63X. Scale bar is 100 um. (**G**) Immunodetection of GLP1R (red), MBP (white) and CASPR (green) imaged with super resolution microscopy at 63X. Scale bar is 100 um Body weight loss (**H**) and cumulative food intake (**I**) in Avp-Cre mice injected with AAV particles expressing hM4Di in the PVH and treated with either DCZ (DCZ PVH^AVPGi^, n=6) or vehicle (DMSO, DMSO PVH^AVPGi^, n=6) and receiving a daily subcutaneous injections with LAGLP1RA (Lira) or vehicle for 7 days. *: p<0.05 Lira vs. vehicle. Data presented as mean ± sem and analysed with 3-way ANOVA. See also Figure S7.

In WT mice, ME uptake of the SAGLP1RA^IR800^ was specifically high in fiber bundles oriented in the rostro-caudal axis (Fig. 6I-6J; Suppl. Video 2; 7A-7B, white arrow), reminiscent of myelinated magnocellular axonal tracks that travel across the dorsal ME to project to the posterior pituitary ^32,11^ (Fig. 7C). Confocal imaging of thin sections from these brains confirmed that SAGLP1RA^IR800^ colocalises with MBP (Fig. 7D, 7D’), indicating that peripherally dosed GLP1R agonists accumulate alongside ME myelinated axons. We obtained further evidence of the neurochemical identity of ME myelinated axons using high-resolution microscopy to colocalise arginine vasopressin (AVP) with MBP and confirmed that vasopressin axons are myelinated, at least in their ME segments (Fig. 7E).

Intriguingly, recent transcriptomic findings from the mouse and human hypothalamus indicate that PVH vasopressin neurons are highly enriched in GLP1R transcript ^9,13^, raising the possibility that peripherally-dosed GLP1R agonists might access PVH GLP1R^+^ neurons through their axonal segments in the ME. To confirm this, we used super resolution microscopy to characterise the expression of the GLP1R in ME myelinated axons. GLP1R immunolabelling overlapped with MBP AVP and CASPR, a protein specifically-expressed at nodes of Ranvier, (Fig. 7F-G, S7A). This indicates that the GLP1R is expressed in the ME, within myelinated axons of magnocellular AVP neurons. Thus, the axonal segment of magnocellular AVP neurons in the ME is a direct target of peripherally-dosed GLP1R agonists.

### PVH vasopressin neurons are required for the weight loss efficacy of GLP1R agonists

The ME portion of PVH GLP1R^+^ axons, which is specifically exposed to GLP1R agonists after peripheral dosing, might represent a functionally relevant site for GLP1R agonists to exert their weight loss effects. To test this, nine-week-old *Avp-Cre* or WT littermates were injected in the PVH with AAV particles expressing cre-inducible hM4Di (PVH^AVPGi^ and PVH^AVP-WT^, respectively), a designer receptor exclusively activated by designer drugs, allowing inducible inhibition of PVH vasopressin neurons with Deschloroclozapine (DCZ), a potent and selective chemogenetic activator ^33^. Immunodetection of the co-expressed RFP reported confirmed spread of the virus throughout the PVH (Fig. S7B). Three-week post viral hM4Di injection, mice were split into 2 groups, treated either with DCZ or its vehicle (DMSO) via the drinking water. After three-week of DCZ or DMSO treatment, mice were dosed subcutaneously with vehicle for 7 days, followed by a 7-day treatment with a LAGLP1RA (liraglutide). Body weight and food intake did not vary between *Avp-cre* mice and WT littermates following hM4Di injection and DCZ or DMSO treatment through the drinking water (data not shown). Likewise, food intake and weight gain remained similar between groups during vehicle treatment. Treatment with the LAGLP1RA significantly reduced appetite and body weight in control PVH^AVP-WT^ mice treated with either DMSO or DCZ, confirming that DCZ treatment itself does not affect these responses. PVH^AVPGi^ mice receiving DMSO through the drinking water also exhibited the expected appetite-suppressing effect and weight loss in response to LAGLP1RA treatment (Fig. 7H, 7I, S7C-S7D). In contrast, the LAGLP1RA failed to produce weight loss and reduce food intake in PVH^AVPGi^ mice receiving DCZ through the drinking water (Fig. 7H, 7I). Thus, PVH vasopressin axons, which access peripherally-dosed GLP1R agonists via their axonal segment in the ME, are required for the weight loss response to GLP1R agonism.

## Discussion

The anti-obesity effect of GIPR agonism occurs primarily via the brain ^3^, yet our understanding of how central activation of the GIPR modulates the neural circuits orchestrating energy homeostasis, and augments weight loss elicited by GLP1R agonism remains incomplete. Here, we show that *Gipr* is expressed in white matter oligodendrocytes and enriched in oligodendrocytes of the ME, highlighting the need to characterise the role of oligodendrocytes in the central action of GIPR activation.

A major finding of our studies is that GIPR signalling bidirectionally regulates oligodendrogenesis in the adult ME. This conclusion is supported by the decrease in new oligodendrocyte production measured in the ME following oligodendrocyte-specific *Gipr* deletion. Conversely, GIPR agonism leads to an increase in the density of *Bmp4*^+^ and BCAS1^+^ oligodendrocytes, two markers specifically expressed in newly-formed and early-myelinating oligodendrocytes^25,26^. Consistent with an increase in oligodendrogenesis, genetic fate mapping studies indicate an increase in the proportion of unlabelled oligodendrocytes (Sox10^+^/Pdgfra^-^/TdTom^-^) in mice treated with the GIPR agonist. Since the GIPR is not expressed in OPCs, this suggests an indirect effect of GIPR activation on OPC differentiation and/or the survival of newly-formed oligodendrocytes. Previous studies have identified a number of signalling molecules secreted by myelinating oligodendrocytes to regulate proliferation and differentiation of local OPCs, for example PDGFA ^34 35^. Alternatively, changes in the survival of mature oligodendrocytes can affect OPC proliferation and differentiation through changes in local myelin debris production, which is a strong regulator of OPCs differentiation ^36^. The increased proportion of Pdgfra^-^/Brdu^+^/Sox10^+^ cells in the ME of GIPRA treated mice following 24hr Brdu labelling further indicates that activation of the GIPR promotes rapid differentiation (loss of PDGFRa expression) of recently divided progenitors, but does not affect proliferation.

While our results indicate increased oligodendrogenesis in response to GIPR agonism in both lean and obese mice, the consequences on oligodendrocyte longevity differ, leading to a difference in the net effect on ME oligodendrocyte and myelin density. In lean mice, GIPR agonism increases ME oligodendrocyte survival, leading to a robust increase in oligodendrocyte density and MBP density, a proxy for myelination. This is remarkable since oligodendrocyte and myelin density in the ME are normally tightly regulated, despite continuous production of new myelinating oligodendrocytes^17^. Together with our data indicating reduced oligodendrocyte survival and MBP density following deletion of the GIPR in oligodendrocytes, these findings support the conclusion that GIPR signalling in oligodendrocytes regulates oligodendrocyte and myelin longevity in the ME. A pro-survival role for GIP-GIPR signalling pathways has been previously reported in pancreatic beta cells through T cell-specific transcription factor (TCF) signalling ^37^. Shared intracellular mechanisms might be engaged in oligodendrocytes, which require TCF7l2 for differentiation and survival ^38^. In contrast, treatment with the GIPR agonist in obese mice promotes oligodendrocyte turnover, suggesting the contribution of distinct cellular pathways in this pathological context. Given the crucial role played by microglia under normal conditions to maintain EM oligodendrocyte and myelin turnover ^17^, this could reflect an impaired phenotype of local phagocytes in the inflamed hypothalamus of DIO mice ^39^. Continuous turnover is a normal feature of ME oligodendrocytes in adult healthy mice ^17^, and while its functional relevance remains to be fully characterised, evidence so far indicates that it benefits energy balance regulation by promoting hypothalamic leptin sensitivity and systemic glucose homeostasis ^28 17^. Thus, the increase in oligodendrocyte turnover during GIPR agonism may help restore blunted oligodendrocyte plasticity in DIO mice, which is a beneficial outcome. These findings highlight the need to investigate the mechanism of action of GIPR-based therapeutics in the obese state, where incretin receptor agonism might engage different pathways.

The consequences of high-fat feeding on oligodendrocyte lineage cells are specific to the ME and not observed in white matter tracts such as the CC ^17^. Likewise, oligodendrocyte and myelin turnover, as well as nutritional regulation of OPC differentiation occur specifically in the ME ^11^. Privileged access to unbuffered circulating factors, including GIPRAs ^8^, might create a local niche in the ME promoting this unique plasticity.

Since whole-body KO of the GIPR confers resistance to diet-induced obesity, we examined the potential contribution of GIPR signalling in oligodendrocytes. As observed following brain wide *Gipr* deletion ^3^, loss of the *Gipr* in oligodendrocytes reduces energy intake but this decrease is compensated by a reduction in energy expenditure, with no change in body weight. Thus, deletion of the GIPR specifically in oligodendrocytes does not lead to the protection against DIO observed following brain wide GIPR deletion. In contrast, unlike brain wide GIPR deletion, lack of the GIPR in oligodendrocytes led to an altered responsiveness to a peripheral insulin bolus, independently of body weight and body composition. This effect is likely mediated by changes in peripheral substrate utilisation and storage, increased lipid deposition in BAT, and a dysregulated BCAA metabolism in OL^GIPR-/-^ mice ^23,40,41^. These results highlight the pleiotropic consequences of adult oligodendrocyte plasticity on systemic metabolism and its contributions to the metabolic consequences of GIPR agonism.

While GIPR agonism enhances the weight loss efficacy of GLP1R agonism in preclinical models of obesity and adult humans with type 2 diabetes, the mechanisms by which activation of GIPR functions improves the outcome of GLP1R signalling in the brain remain to be elucidated. Importantly, transcriptomics studies have failed so far to identify a candidate appetite-suppressing cell population co-expressing the GIPR and GLP1R, a fortiori in brain areas accessed by incretin-based therapeutics ^8 30^, suggesting a multi-cellular mode of action ^7,9,10^. Most studies aiming to elucidate the mechanism through which GIPR agonism improves the efficacy of GLP1R agonists have focused on identifying the neuronal populations and/or downstream circuits promoting the synergistic appetite suppression. GABAergic neurons of the area postrema ^42 15^ have been proposed to contribute by attenuating the aversive response to GLP1R agonism. Further, brain-wide deletion of the GIPR in GABA-ergic neurons suppresses the potentiating action of GIPR agonism on the weight loss response to GLP1R agonism ^43^. As expected, deletion of the GIPR in GABAergic neurons blunts neuronal activation in the area postrema following treatment with a GIPR agonist ^43^. However, other GABA-ergic GIPR-expressing populations, such as in the hypothalamus ^7^, might also contribute to this phenotype. Here, we propose that activation of the GIPR in oligodendrocytes contributes to the synergistic weight loss provide by incretin-based multi-receptor agonists. Our results provide evidence that differential brain access to relevant GLP1R target sites, here the GLP1R-expressing cell compartments of the ME, is a mechanism through which GIPR agonism can enhance the weight loss efficacy of GLP1R agonists. We show that GIPR agonism increases the expression of *Vegfa* and VEGF in the median eminence of HF-fed mice. VEGF is a well-known potent inducer of vascular hyperpermeability ^44,45^. In the ME, VEGF expression has been shown to upregulate ME-ARH barrier permeability and hormone access through the regulation of local vessel fenestration ^46,47^. Consistently, we observed an increase in the density of fenestrated capillaries in the median eminence of mice treated with GIPRA Our data convincingly show that increased diffusion of the fluorescently-labelled GLP1R agonist in the ME-ARH requires activation of the GIPR in oligodendrocytes. We propose that the increase in new oligodendrocyte production following GIPRA treatment regulates barrier function in response to GIPR agonism. In fact, newly-formed oligodendrocytes have been recently implicated in the regulation of vascular permeability in the ME through the regulation of VEGF-A expression ^27 28^. Collectively, these data support the conclusion that GIPR signalling in oligodendrocytes increases the permeability of the ME-ARH barrier and may facilitate increased access of incretin therapies to anorectic neuronal populations expressing the GLP-1R.

A key finding of our studies is that PVH^AVP^ neurons are implicated in the weight loss efficacy of systemic GLP1R agonism. This finding is particularly noteworthy given recent reports indicating that PVH^AVP^ neurons represent one of the neuronal populations with highest GLP1R enrichment in the mouse and human hypothalamus ^9,13^. Importantly, a role for PVH^AVP^ neurons in the control of food intake and the long-term regulation of body weight is highlighted by numerous studies in preclinical models of obesity ^48,49-51.^ Further, PVH^Glp1-r^ neurons are important for the control of acute feeding behaviour and energy homeostasis ^52,53^. However, our results contrast with the lack of effect of acute chemogenetic inhibition of PVH^Glp1-r^ neurons on liraglutide-induced appetite suppression ^53^, which suggests that alternative mechanisms might be engaged, at least acutely. Importantly unlike most PVH neuronal populations, PVH^AVP^ neurons receive virtually no inputs from preproglucagon neurons and therefore might not be relevant to the signalling of brain-derived GLP1 ^54^. Instead, GLP1R in PVH^AVP^ neurons might be designed to specifically respond to peripheral GLP1. How circulating GLP1R agonists reach PVH^AVP^ neurons is unclear, since these molecules do not cross the blood-brain-barrier and the PVH is distal from brain sites where their free diffusion occurs ^31 30^. Here we show that the GLP1R is enriched in the axons of magnocellular neurons, at least at the level of the ME, creating a privileged site of access to peripherally injected GLP1R agonists for these neurons. Consistently, we observed the accumulation of fluorescently labelled GLP1R agonists in ME axons following a peripheral administration.

In summary, our findings identify a novel mechanism by which incretin therapies function to promote synergistic weight loss in the management of excess adiposity.

### Limitations of Study

The model used to delete *Gipr* from oligodendrocytes only achieved partial deletion. Future work deleting *Gipr* from the entire oligodendrocyte lineage might uncover additional roles for GIPR signalling in oligodendrocytes in energy and glucose homeostasis. In the model used in this study, although deletion of *Gipr* in oligodendrocytes did not produce a marked change in metabolic phenotype, we did observe mild changes which might have altered the amplitude of the responses to incretin receptor agonists during the weight loss intervention.

## Method Details

### Tamoxifen preparation and administration

Tamoxifen was prepared in corn oil by sonication at 37 °C at 30 mg/ml prior to administration by oral gavage at 300 mg/kg on 4 consecutive days in *Opalin-Cre/ERT2;Ai9* mice, or through the intraperitoneal at 80 mg/kg for 8 consecutive days in *Plp-Cre/ERT2* mice.

### GLP1R and GIPR agonist preparation and administration

The LAGIPRA, LAGLP1RA and SAGLP1RA^IR800^ were synthesized at Eli Lilly and Company, dissolved in 40mM Tris-HCl pH8 with 0.02% PS-80, and dose subcutaneously or intravenously at 300nmol/kg, 100nmol/kg and 100nmol/kg, respectively. LAGIPRA, LAGLP1RA were dosed at ZT8-ZT9. Before SAGLP1RA^IR800^ administration, mice were fasted for 4h. The GIPRA^cy5^ was prepared a previously described ^8^ and dosed intravenously at 100nmol/kg.

### EdU chase experiment

Mice were administered EdU through 4 intraperitoneal injections (50 mg/kg; prepared in sterile saline at 5 mg/ml; 1 injection every 6h) during the 24h preceding terminal perfusion.

### Brain tissue preparation

Animals were anaesthetized with an ip injection of 50 ul pentobarbitol (Dolethal, 200 mg/ml) then transcardially perfused as follows. For immunohistochemistry (IHC), animals were perfused with 0.01 M phosphate buffered saline (PBS) at room temperature (RT) followed by 4% paraformaldehyde in PBS (pH 7.4) at 4°C. For clearing experiments, animals were perfused with 0.01 M phosphate buffered saline (PBS) at room temperature (RT) followed 10% NBF. For RNA-scope experiments, the brain is collected and quick frozen on crushed dry ice and stored at - 80°C until further use.

### Fluorescent in situ hybridization

Multiplexed fluorescent in situ hybridizations (ISH) in brain slices were conducted using RNAscope technology (Advanced Cell Diagnostics, Newark CA). Each brain was divided into forebrain and hindbrain by a coronal cut at the level of the pons and mounted on the precooled cryostat holder with Tissue-Tek O.C.T. compound (Sakura Finetek). 12 µm thick coronal sections covering the 1) ME/ARH 2) VTA/SN 3) AP/NTS and 4) spinal cord/dorsal horn were collected on SuperFrost Plus microscope slides. ISH was performed on tissue sections using the RNAscopeTM Multiplex Fluorescent V2 Assay (Advanced Cell Diagnostics, Cat. 323100) to simultaneously visualize up to four different mRNAs using target specific probes. Appropriate negative and positive controls were included and run in parallel. RNAscope assay was performed according to the manufacturer’s user manual. After ISH, sections were counterstained with DAPI and coverslipped using a fluorescence mounting medium. Finally, slides were scanned under a 20X objective in an Olympus VS-120 slide scanner with appropriate fluorescent filters. Densities of cells per mm2 were obtained for 3 sections per animal.

### Immunofluorescence

Brains were post-fixed overnight at 4 °C in 4% PFA then cryoprotected in 30% (w/v) sucrose (Fisher Scientific) solution in PBS for at least 48 h prior to processing. Tissues were covered with Optimal Cutting Temperature (OCT) medium (CellPath, Newtown, UK) and sections were obtained at 25 μm on a Leica SM2010R Freezing Microtome (Leica, Wetzlar, Germany). All sections were subjected to heat-mediated antigen retrieval in 10 mM sodium citrate (pH6.5; Fisher Scientific) in distilled water for 20 min at 80 °C prior to washing 3 times in PBS. For all experiments, sections were blocked in normal donkey serum (NDS, Vector Biolabs, Philadelphia, Pennsylvania) diluted in PBS containing 0.3% Triton X-100 (0.3% PBST; Sigma) for 1 h prior to primary antibody incubation overnight at 4 °C. Following primary antibody incubation, sections were washed 3 time with 0.1% PBST and incubated with appropriate fluorophore-conjugated secondary antibodies diluted 1:500 in 0.3% PBST for 2 h at room temperature. Sections were subsequently washed with 0.1% PBST and mounted to Clarity microscope slides (Dixon Science, Edenbridge, UK) under coverslips (1.0 thickness; Marienfeld, Lauda-Königshofen, Germany) with Vectashield Vibrance Mounting Medium with 4′,6-diamidino-2-phenylindole (DAPI; Vector Laboratories, Newark, California). Alexa405, Alexa488, Alexa555, Alexa594 and Alexa647 conjugates (Life Technologies, Carlsbad, California) were used as secondary antibodies.

### Tissue Clearing

Brains were dissected and immersion fixed in NBF overnight at room temperature. The samples were then washed 3×30 minutes in PBS with shaking. Tissue was dehydrated in MeOH/H2O series: 20%, 40%, 60%, 80% and 100%, for 1 hour each, at room temperature. Samples were incubated in 100% MeOH overnight and the next day for 3 hours (with shaking) in 66% DCM/33% MeOH at room temperature and in 100% DCM for 15 minutes twice (with shaking) to remove traces of methanol. The samples were finally transferred to Dibenzyl Ether (DBE) and stored in closed glass vials in the dark.

### Light-sheet Imaging

All samples were imaged using a Lavision ultramicroscope system II and MV PLAPO 2X C objective. Whole mouse brains were imaged at 1.26 × magnification. In addition, the medio basal hypothalamus was imaged at 4 x magnification. The fluorescent signal was captured at the autofluorescence channel (560nm) and compound-specific channel (790nm). DBE is used as a clearing agent during the acquisition of data. The Imaris software was used to visualize the data in 3D.

### Confocal microscopy

For all experiments, slides and images were blinded to experimental condition. Sections were imaged with a Leica SP8 confocal microscope using either a 40× or 63× oil objective. Sections were imaged as z-stacks at intervals of 0.3 μm with tile scanning to obtain signal from the entire depth and area of the region of interest (ROI). Microscope settings were identical for image acquisition within each experiment. Images were analysed using Fiji software.

### Super-resolution microscopy

Following standard immunohistochemistry procedure described above and the wash step of the secondary antibody, samples were incubated in Hoechst 33342 (1:10,000 in PBS, Invitrogen) for 5 minutes, then washed three times for 5 minutes each in PBS. Sections were subsequently mounted onto Clarity microscope slides (Dixon Science) using 1.5H coverslips (Carl Zeiss) and ProLong Diamond Mounting Medium (Thermo Fisher). The slides were left to dry at room temperature overnight before being stored long-term at 4°C. Imaging was performed using an LSM 980 confocal microscope (Zeiss) equipped with Airyscan. Appropriate long-pass (LP) and band-pass (BP) filters were applied during acquisition to minimize channel bleed-through. Initial Airyscan post-processing was conducted using Zen Black software (Zeiss), with further image processing and export performed in Fiji (ImageJ). All imaging conditions were maintained identically between slides.

### Image analysis

Images obtained from cleared brains using light sheet microscopy were analysed as previously described ^31^. Images obtained from RNAscope experiments were imported into the VIS software (Visiopharm, Denmark) for ah-hoc analysis. Images acquired using confocal microscopy were processed as follows. Three or more sections per animal were used for quantification. All confocal images were analysed using Fiji software. Prior to analysis, z-stacks were projected into a single image and the image scale calibrated to determine cell density (number of cells/unit area). The area of ROIs were calculated by tracing the ROI with the freehand tool and measuring - all ROI borders were determined using the Paxinos and Franklin Mouse Brain Atlas. Cells labelled for specific markers, alone or in combination with others, were counted using the Manual Cell Counter Fiji Plugin. The identity of each biological sample was revealed once all analysis for a given experiment was complete.

### Quantitative polymerasechain reaction

Fresh hypothalami were collected into RNALater solution (ThermoFisher) and stored at −20 °C until processing. RNA was extracted from tissues samples using an RNEasy Micro Kit following the manufacturer’s protocol. 100–200 ng of total RNA was reverse transcribed to cDNA using the High Capacity cDNA Reverse Transcription Kit) according to the manufacturer’s instructions. Gene expression was assessed using either SYBR Green or TaqMan technologies on a QuantStudio 5 (Applied Biosystems). Relative gene expression was calculated by the 2ˆ(-ΔΔCt) method. Data were normalised to the housekeeping gene *Gapdh,* as the expression of this gene did not change between groups. All primers and probes were obtained from Sigma–Aldrich and commercially available TaqMan assays were obtained from Thermo Fisher.

### Metabolic phenotyping

Body composition was analysed using a EchoMRI Whole Body Composition Analyser (EchoMRI, Houston, Texas). Promethion High-Definition Multiplexed Respirometry Cages (Sable Systems International, Las Vegas, Nevada) were used to analyse energy expenditure by indirect calorimetry, food intake, water intake, respiratory quotient and activity over 48 h in mice single-house for at least one week prior. Data collected during the first 24 h of each run was discarded to allow for acclimatisation of mice to the altered cage environment.

### Glycemic control phenotyping

For the oral glucose tolerance test, mice were food deprived for 6 hr and blood was sampled from tail vein immediately prior to glucose bolus (gavage, 2 mg/kg), and 10, 20, 30, 60, 90, and 120 min following bolus administration. For the insulin sensitivity test, mice were food deprived for 6 hr and blood was sampled from tail vein immediately prior to the administration of an insulin bolus (ip, 0.75U/kg), and 10, 20, 30, 60, 90, and 120 min following bolus administration. Blood glucose was analysed using an AlphaTrak3 handheld glucometer (Precision Xtra; MediSense).

### Stereotaxic surgery, viral injections and chemogenetic studies

Surgical procedures were conducted on 9- to 11-week-old male AVP-Cre and wild-type littermate mice. All animals received Metacam prior to surgery and 24 h after surgery. Under isoflurane-induced anesthesia, 300nL AAV8-hSyn-DIO-hMD4(Gi)-mCherry (Titer 5 x 1012 genomic copies; University of North Carolina Viral Core facility, USA) were injected into the PVH (A/P: -0.70 mm, D/V: -4.75 mm, and lateral: +/− 0.25 mm from the bregma) with a bilateral steel guide cannula and a 33-gauge stainless steel injector (Plastics One). Following the surgery, mice were allowed for a 3 week recovery. After that, 5mg/ml deschloroclozapine (DCZ) or the same volume of vehicle DMSO were introduced to the drinking water and refreshed twice a week throughout the rest of study. Two weeks after the DCZ or DMSO introduction, the mice were single-housed for 1 week, following by daily subcutaneous injections of saline (50mL/kg) during ZT 8-10 for 1 week to obtain an individual baseline response to the procedures and then daily subcutaneous injections of liraglutide (0.2mg/kg; Tocris, UK) during the same time-of-day for another week. Food and animals were weighed daily during these 2 weeks. After the last treatment, mice were allowed to recover for 1 week while being maintained on DCZ- or DMSO-added drinking water. Mice were perfused with 4% PFA transcardially after the terminal treatments as indicated.

### Oil Red O Staining

PFA-fixed tissues were cryoprotected in 30% sucrose solution (w/v in distilled water; Sigma) overnight before sectioning at 8-10μm on a Leica CM1950 cryostat onto electrostatically charged slides. A stock solution of ORO was made up by gently heating 0.5 g ORO (Sigma) in 100 ml absolute isopropyl alcohol (Sigma) in a water bath overnight. A working solution was prepared by mixing 60 ml ORO stock solution and 40 ml 1% dextrin (Fisher Scientific) in distilled water. The working solution was allowed to stand for one day and then filtered. Slides were rinsed in PBS prior to incubation with ORO working solution for 20 minutes. Excess stain was rinsed off with distilled water and sections counterstained with haematoxylin for 20 seconds and blued in tap water. Coverslips were mounted with Pertex mounting medium (Pioneer Research Chemicals Ltd.). Images were acquired with an Axioscan Z1 Slide Scanner (Zeiss) using a 20x objective and analysed using the vacuoles module on HALO Image Analysis Software.

### Targeted Metabolomics

Targeted metabolomics was performed on plasma, liver, interscapular brown adipose tissue (iBAT), epididymal white adipose tissue (eWAT) and muscle samples using a liquid chromatography with tandem mass spectrometry (LC-MS/MS) approach, as described in ^55^. Briefly, plasma samples were frozen immediately after collection and then thawed in an ice bath prior to LC-MS/MS analysis. Two extraction protocols (methods A and B) were used to collect metabolomics data. For method (A), 150 μL of pre-chilled acetonitrile-methanol (1:1 v/v) solvent mixture was added to 25-μL plasma aliquot. For method (B), 150 μL of pre-chilled methanol-water (4:1 v/v) solvent mixture was added to a second 25-μL plasma aliquot. Samples were mixed thoroughly and incubated at −20°C overnight. Both extraction solutions were spiked with a mix of stable labelled internal standards. After overnight extraction, the samples were centrifuged for 15 minutes at 14000 g at +4°C. A pooled calibrator sample was created for each method by combining an aliquot of supernatant from all the extracted samples together. The pooled calibrator was serially diluted in the same extraction solvent and used to create a calibration curve (100% [pooled calibrator], 75%, 50%, 30%, 15%, 10%, and 5%) for relative quantitation and batch-to-batch data normalization. Individual samples were further diluted at 1:1 (v/v) ratio with each extraction solution, transferred into a new 96-well plate, and injected for LC-MS/MS analysis.

Tissue samples were flash frozen in liquid nitrogen immediately after collection and stored at −80 °C. Frozen tissue samples were pulverized using a tissue pulverizer and kept frozen until LC-MS/MS analysis. Two aliquots of pulverized tissue were weighted for each sample to run methods A and B independently. Tissue samples were extracted and processed as described above. Extraction solutions were added to reach a final concentration of 100 mg/mL.

Data were acquired using a Shimadzu Nexera X2 UPLC system coupled to an AB Sciex 6500+ triple quadrupole mass spectrometer equipped with an electrospray source. For method A, a Waters Acquity BEH Amide 100 mm x 2.1 mm, 1.7 mm particle size, column was used; for method B, a Waters XSelect HSS T3 C18 100 mm x 2.1 mm, 1.8 mm particle size, column was used. Both columns were maintained at +40C. Elution solvents for both methods were 10 mM ammonium formate adjusted with 0.1% formic acid (solvent A) and 0.1% formic acid in acetonitrile (solvent B). Data were acquired using scheduled multiple reaction monitoring mode with polarity switching. A total of 250 polar metabolites were targeted. For most of the metabolites, identification was supported by 1 qualifier ion monitored in addition to a quantifier ion. Two qualifier ions were monitored to resolve interferences amongst few metabolites (data not shown). Peak areas were integrated using the AB SCIEX MultiQuant 3.0.2 software. Only analytes detected at the lowest pooled quality control calibrator with signal-to-noise >3 and detected in more than 75% of the individual samples were quantified. Relative quantitation to the pooled calibrators was achieved using a linear regression model. Metabolite areas were normalized to internal standard area responses. For analytes with no matching stable labelled internal standards, the optimal choice (picked amongst the internal standards monitored under the same polarity and within the same assay) was the internal standard giving the minimum root mean square error for the pooled calibrators and best linear fit. Individual sample values falling below the limit of detection were imputed to 1/2 times the lower calibrator.

For statistical analysis, the metabolomics data were log-transformed. On-way ANOVA with Dunnett’s post hoc test was performed to assess significance (p < 0.05) of between-group data distributions for individual metabolites. Multivariate analysis of variance (MANOVA) was also performed on principal components arising from principal component analysis (PCA) to assess changes in metabolomic profile comprehensively. Spearman’s rank correlations were calculated to explore the association between metabolites. DiffCorr package was used to identify pattern changes in correlation networks (Fukushima 2013). Statistical analyses were implemented by using Matlab R2019a (Mathworks) and R 3.6.0.

### Statistical analysis

All data visualisation and statistical analysis was performed in Prism 9 Software (GraphPad). or all statistical tests, an α risk of 5% was used. All kinetics were analysed using repeated-measures two-way ANOVAs and adjusted with post hoc tests. Multiple comparisons were tested with one-way ANOVAs and adjusted with Tukey’s post hoc tests. Single comparisons were made using two-tail Student’s t tests.

## Supplementary Material

Supplemental information

Supplementary Table S1

Supplementary Table S2

Supplementary Table S3

## Data Availability

All data and code to understand and assess the conclusion of this research are available in the main text and supplementary materials. Data used to generate the figures are available in Data S1.

## Highlights

- GIPR signalling in oligodendrocytes regulates adult median eminence oligodendrogenesis
- GIPR signalling in oligodendrocytes is required for GIPR activation to improve the weight loss efficacy of GLP1R agonism
- GIPR agonism increases brain access of GLP1-R agonists to the mediobasal hypothalamus
- Vasopressin neurons of the paraventricular nucleus of the hypothalamus access peripherally dosed GLP1R agonists through their axonal compartment in the median eminence, and are required for GLP1R agonists induced weight loss
