# Supplementary material for: Glucose-dependent insulinotropic polypeptide receptor signalling in oligodendrocytes increases the weight loss action of GLP-1R agonism
Source: Cell Metab. Author manuscript; Available in PMC 2026 Sep 4. (PMC7619446; doi:10.1016/j.cmet.2025.07.009)
Supplement: Supplementary file 1 [file EMS211979-supplement-Supplemental_information.pdf]

Cell Metabolism, Volume *37*

Supplemental information

Glucose-dependent insulinotropic polypeptide receptor signaling in oligodendrocytes increases the weight-loss action of GLP-1R agonism

Robert Hansford, Sophie Buller, Anthony H. Tsang, Simon Benoit, Anna G. Roberts, Emmy Erskine, Thomas Brown, Valentina Pirro, Frank Reimann, Norio Harada, Nobuya Inagaki, Ricardo J. Samms, Johannes Broichhagen, David J. Hodson, Alice Adriaenssens, Soyoung Park, and Clemence Blouet

Supplementary Information

Figure S1: The *Gipr* is enriched in murine and human ME mature oligodendrocytes, related to figure 1. Representative images **(A)** and quantifications **(B)** of FISH detection of *Gipr* (green) in oligodendrocyte lineage cells using *Sox10* to label the entire lineage (white), *Pdgfra* (Ra, red) to label OPCs, and *Plp1* (yellow) to label oligodendrocytes in the mouse brain. Scale bar is 50um. Red arrow heads: OPCs, Yellow arrow heads: *Gipr*$^{-}$ oligodendrocytes, white arrow heads: *Gipr*$^{+}$ oligodendrocytes. Representative images **(C)** and quantifications **(D)** of FISH detection of *Gipr* (green) in oligodendrocyte lineage cells using *Sox10* to label the entire lineage (white), and *Plp1* (yellow) to label oligodendrocytes in the mouse brain. Scale bar is 50um. Green arrow heads: *Gipr*$^{+}$ oligodendrocytes. Data are presented as mean ± sem.

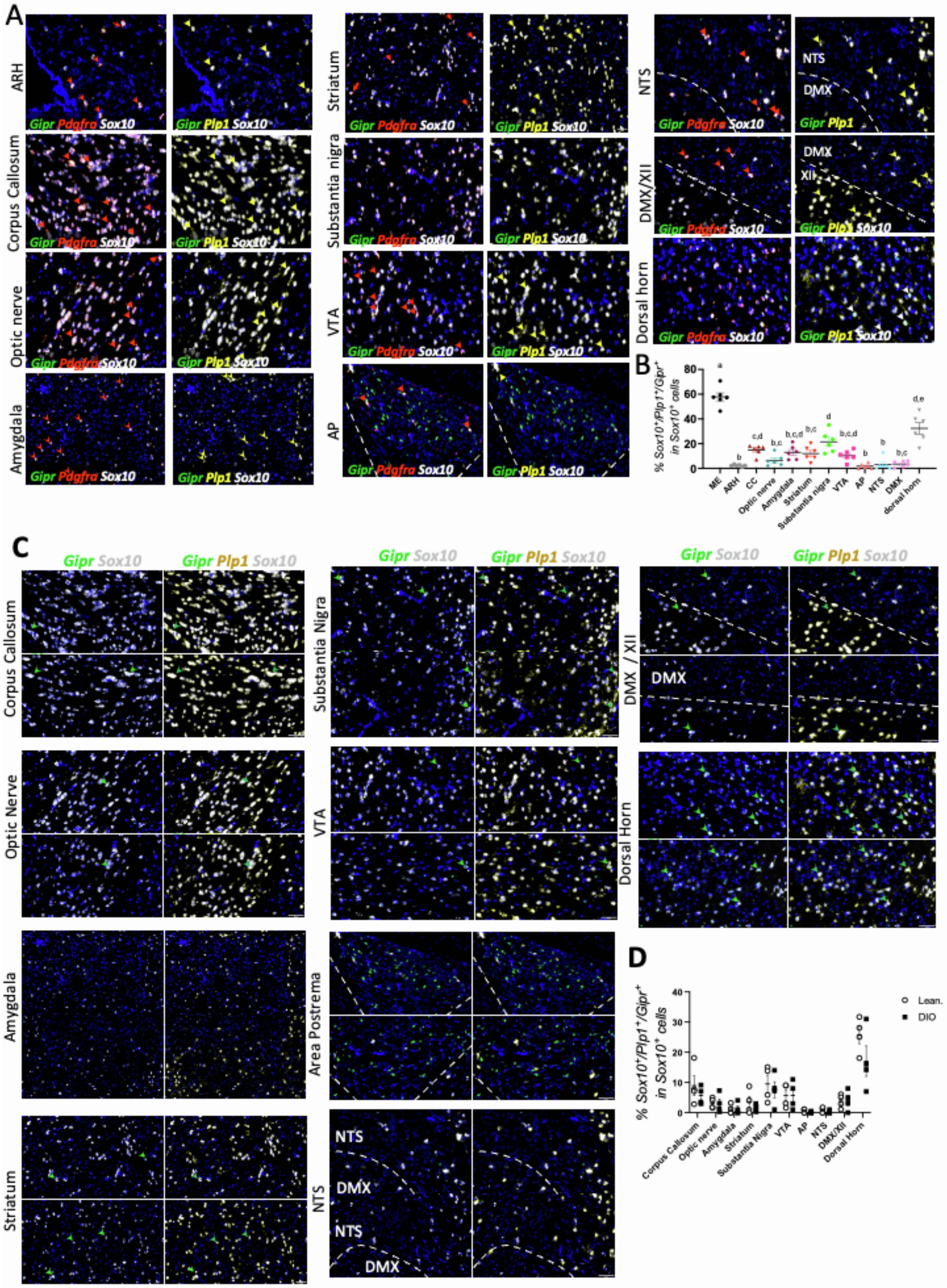
A
ARH
Corpus Callosum
Optic nerve
Amygdala
Striatum
Substantia nigra
VTA
AP
NTS
DMX
DMX/XII
XII
Dorsal horn
Gipr Pdgfra Sox10
Gipr Plp1 Sox10
Gipr Plp1
B
% Sox10+/Plp1+/Gipr+ in Sox10+ cells
ME
ARH
CC
Optic nerve
Amygdala
Striatum
Substantia nigra
VTA
AP
NTS
DMX
dorsal horn
C
Gipr Sox10
Gipr Plp1 Sox10
Corpus Callosum
Optic Nerve
Amygdala
Striatum
Substantia Nigra
VTA
Area Postrema
NTS
DMX / XII
DMX
Dorsal Horn
D
% Sox10+/Plp1+/Gipr+ in Sox10+ cells
Lean.
DIO
Corpus Callosum
Optic nerve
Amygdala
Striatum
Substantia Nigra
VTA
AP
NTS
DMX/XII
Dorsal Horn

Figure S2: GIPR signalling in oligodendrocytes regulates adult ME oligodendrogenesis and myelination in HF-fed mice, related to figure 2. (A) MBH *Gipr* mRNA expression measured by qPCR and (**B**) body weight gain on a 60% HF diet in $OL^{GIPR\,+/+}$ and $OL^{GIPR\,-/-}$ mice. **(C)** FISH detection of *Gipr* (green) in oligodendrocytes using *Plp1* to label the myelinating oligodendrocytes (red) in brain sections from $OL^{GIPR+/+}$ and $OL^{GIPR-/-}$ mice. Scale bar is 100um/ Representative images **(D)** and quantification **(E)** of oligodendrocyte lineage cells labelled with YFP at P60 for fate-mapping purposes and detected using multiplexed immunofluorescent detection of SOX10 (red), PDGFRa (Ra, white), and YFP (green) in the corpus callosum of HF-fed $OL^{GIPR\,+/+}$ and $OL^{GIPR\,-/-}$ mice. Scale bar is 50µm. Data are presented as mean ± sem.

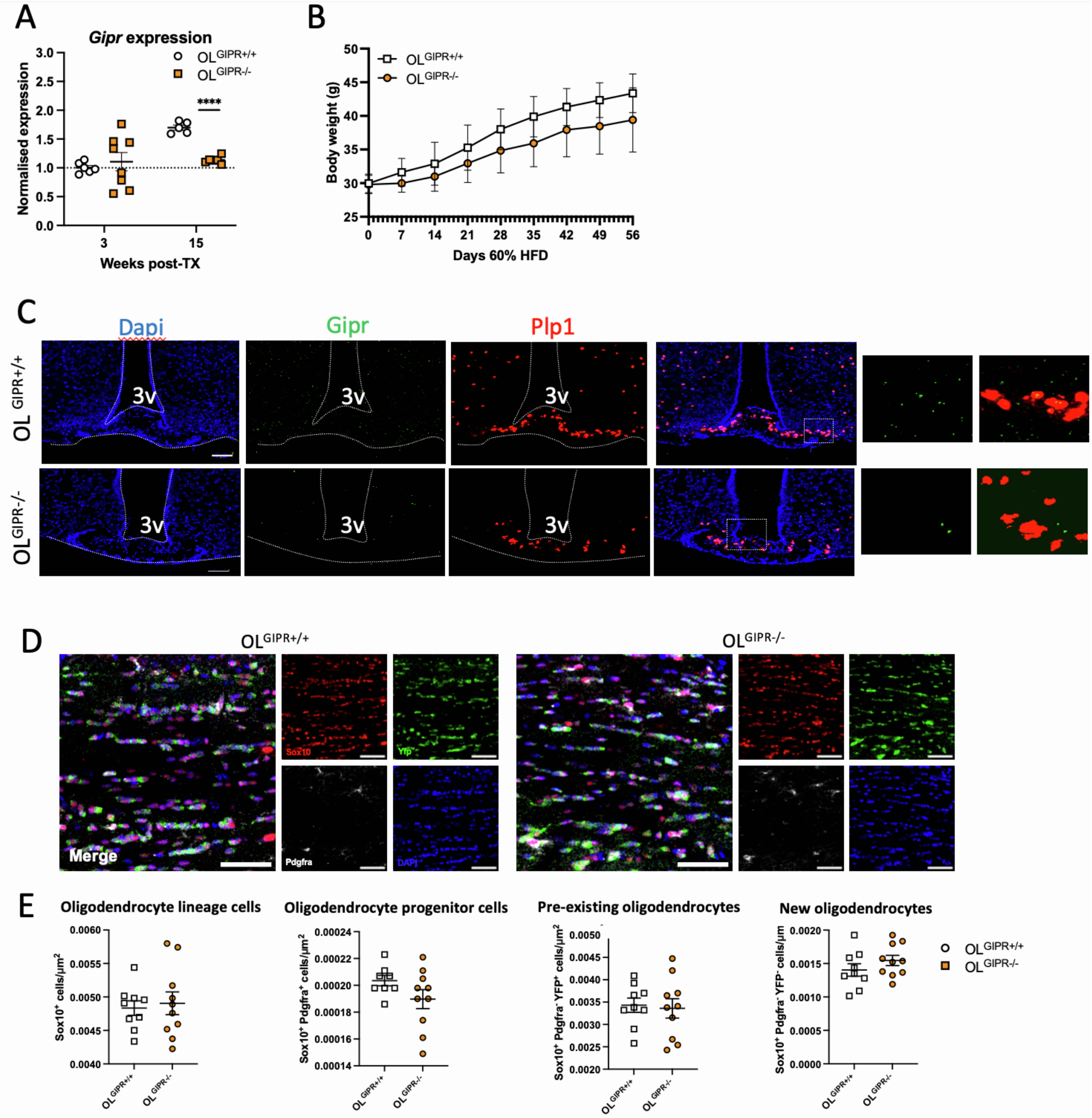

Figure S3: GIPR signalling in oligodendrocytes regulates energy expenditure and insulin sensitivity in HF-fed mice, related to figure 3. Respiratory quotient (RQ, **A**), locomotor activity (**B**), epididymal white adipose tissue (eWAT, **C**) and liver cellularity analysis (**D**), MANOVA analysis of metabolomics data from plasma and eWAT (**E**), and eWAT and iBAT concentrations of key BCAA metabolites (**F**) in OL$^{GIPR-/-}$ (n=6) and OL$^{GIPR+/+}$ (n=6) mice. Scale bar is 50um. Data are presented as mean ± sem.

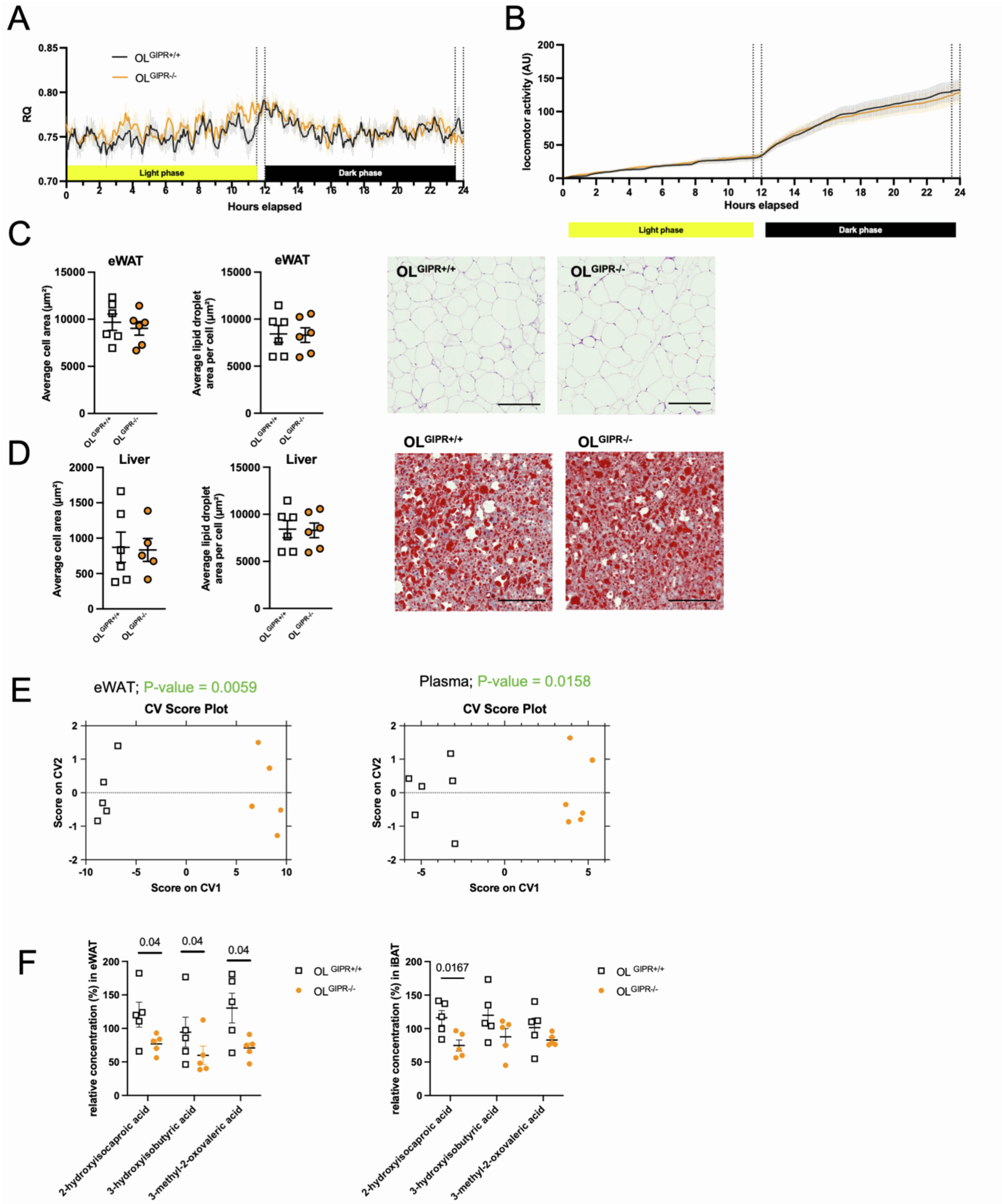

Figure S4: GIPR agonism regulates ME oligodendrogenesis and myelin plasticity, related to figure 4. (**A**) Study design. (**B, D**) Body weight gain in chow-fed (**B,C**) and HF-fed (**B, D**) *Opalin-iCreER*^{T2}*:TdTom* mice following Tm administration at P60 and 2 weeks of subcutaneous daily dosing with a GIPR agonism of vehicle. Representative images (E) and quantification (F) of FISH detection of *Bmp4* (green) in oligodendrocyte lineage cells using *Sox10* to label the entire lineage (white)in the mouse ME, arcuate nucleus (ARH), area postrema (AP) and nucleus of solitary tract (NTS). Data are presented as mean ± sem.

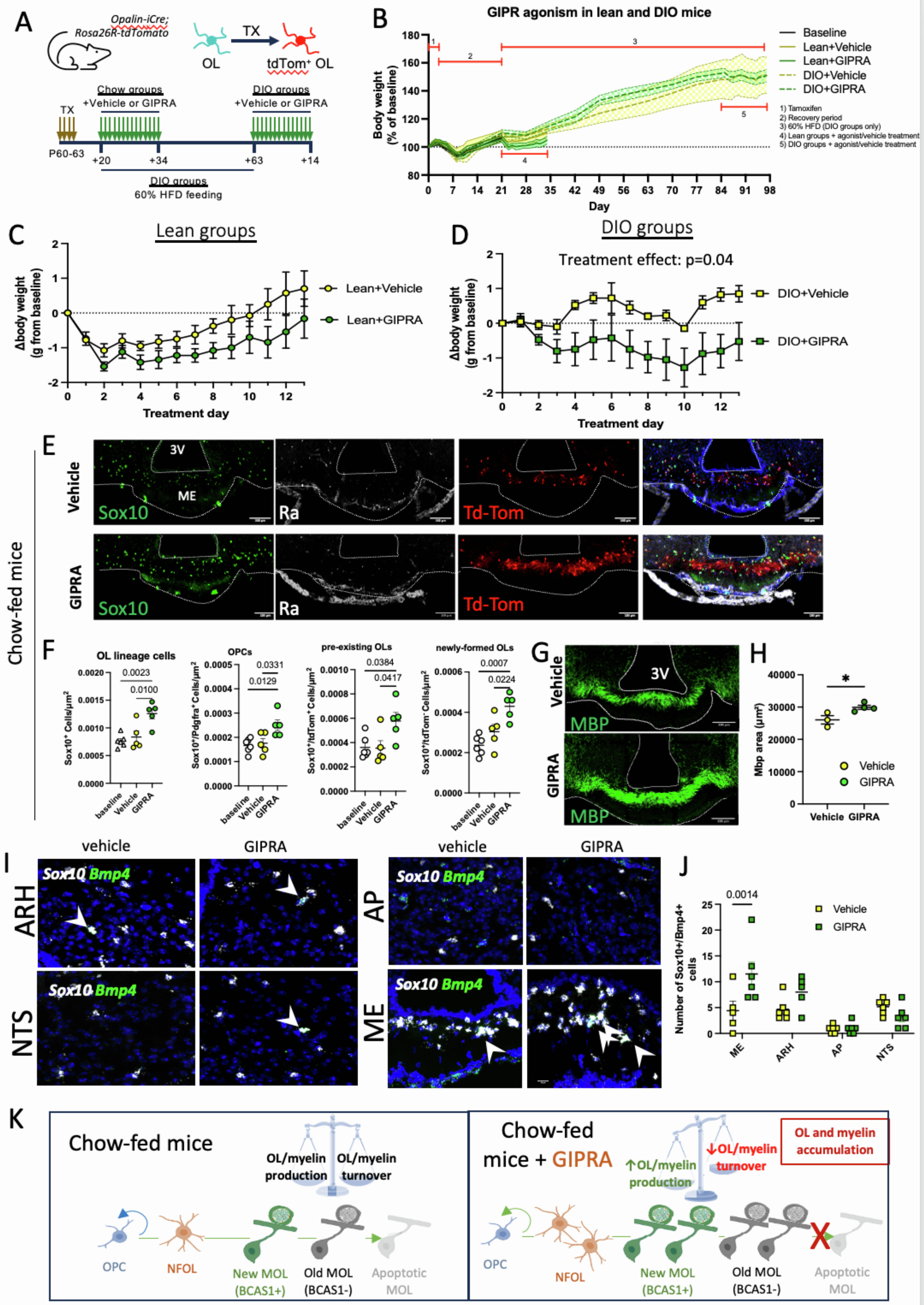

Figure S5: GIPR signalling in oligodendrocytes is required for the beneficial effects of GIPR agonism the anti-obesity actions of GLP-1R agonist, related to figure 5. (**A**) Experimental design of study used to establish the response to incretin receptor agonists in $OL^{GIPR+/+}$ and $OL^{GIPR-/-}$ mice. (**B**) Body weight gain in $OL^{GIPR+/+}$ and $OL^{GIPR-/-}$ mice during the development of DIO. Data are presented as mean ± sem.

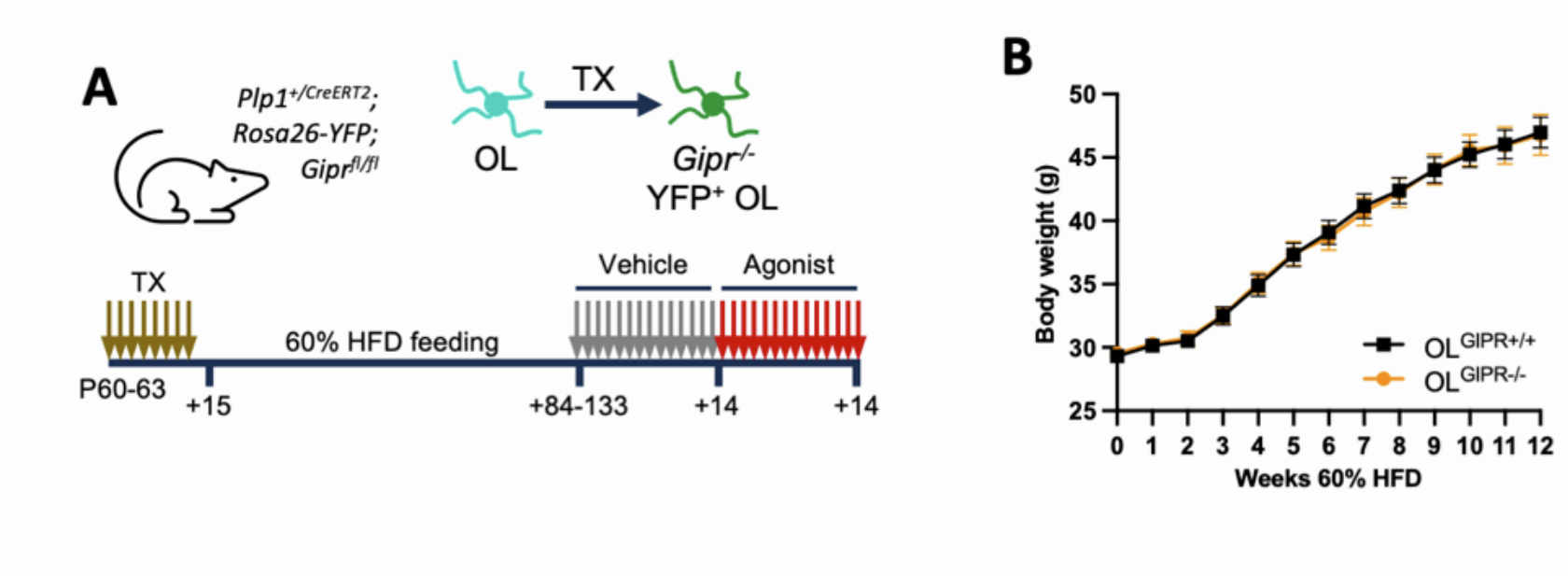

Figure S6: Distribution of labelled short-acting GLP-1R agonist in the mouse brain following intravenous administration, related to figure 6. (A) Image showing group changes in IR800 fluorescence between the Vehicle and SAGLP-1RA^{IR800} treated groups (n=6). Brain regions where the IR800 fluorescence is increased in the SAGLP-1RA^{IR800} group compared to the vehicle group are shown in blue. Scale bar 500mm. (**B**) Bar chart demonstrating the top 20 brain regions that are significantly upregulated in the SAGLP-1RA^{IR800} group compared to the vehicle group, ranked by significance. *: P<0.05, **: P<0.01, ***: P<0.001 (**C**) experimental design and (**D**) Body weight gain in HF-fed OL$^{GIPR-/-}$ mice and WT littermates following a pre-treatment with vehicle or GIPR agonism for 7 days and a terminal bolus with IR800-Ex4. Data are presented as mean ± sem.

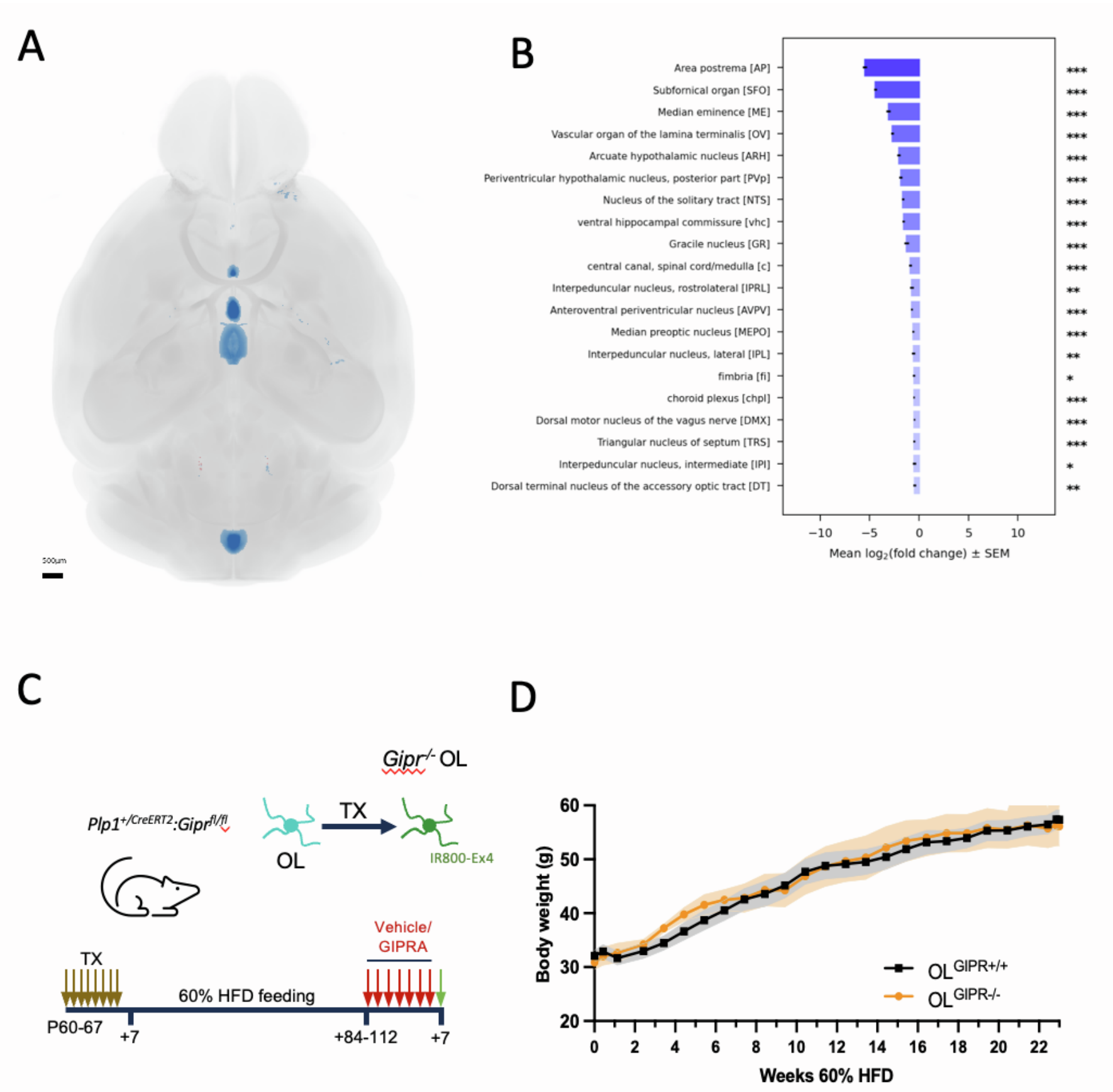

Figure S7: Distribution of labelled short-acting GLP-1R agonist in the mouse brain following intravenous administration, related to figure 7. (A) Validation of the GIPR antibody in brain tissues from GLP-1R KO mice. (**B**) Image showing group changes in IR800 fluorescence between the Vehicle and SAGLP-1RA^{IR800} treated groups (n=6). Brain regions where the IR800 fluorescence is increased in the SAGLP-1RA^{IR800} group compared to the vehicle group are shown in blue. Scale bar 500mm. (**B**) Bar chart demonstrating the top 20 brain regions that are significantly upregulated in the SAGLP-1RA^{IR800} group compared to the vehicle group, ranked by significance. *: P<0.05, **: P<0.01, ***: P<0.001. (**D**) Representative image of RFP (red) and AVP (green) expression in the PVH of *Avp-Cre* mice 3 weeks after a PVH injection of AAV hSyn-DIO-hM4D(Gi)-mCherry. Body weight change (**E**), and cumulative food intake (**F**) in AVP-WT mice injected with AAV particles expressing hM4Di in the PVH and treated with either DCZ (DCZ PVH^{AVPGi}, n=4) or vehicle (DMSO, DMSO PVH^{AVPGi}, n=3) and receiving a daily subcutaneous injections with LAGLP-1RA (Lira) or vehicle for 7 days. Data are presented as mean ± sem.

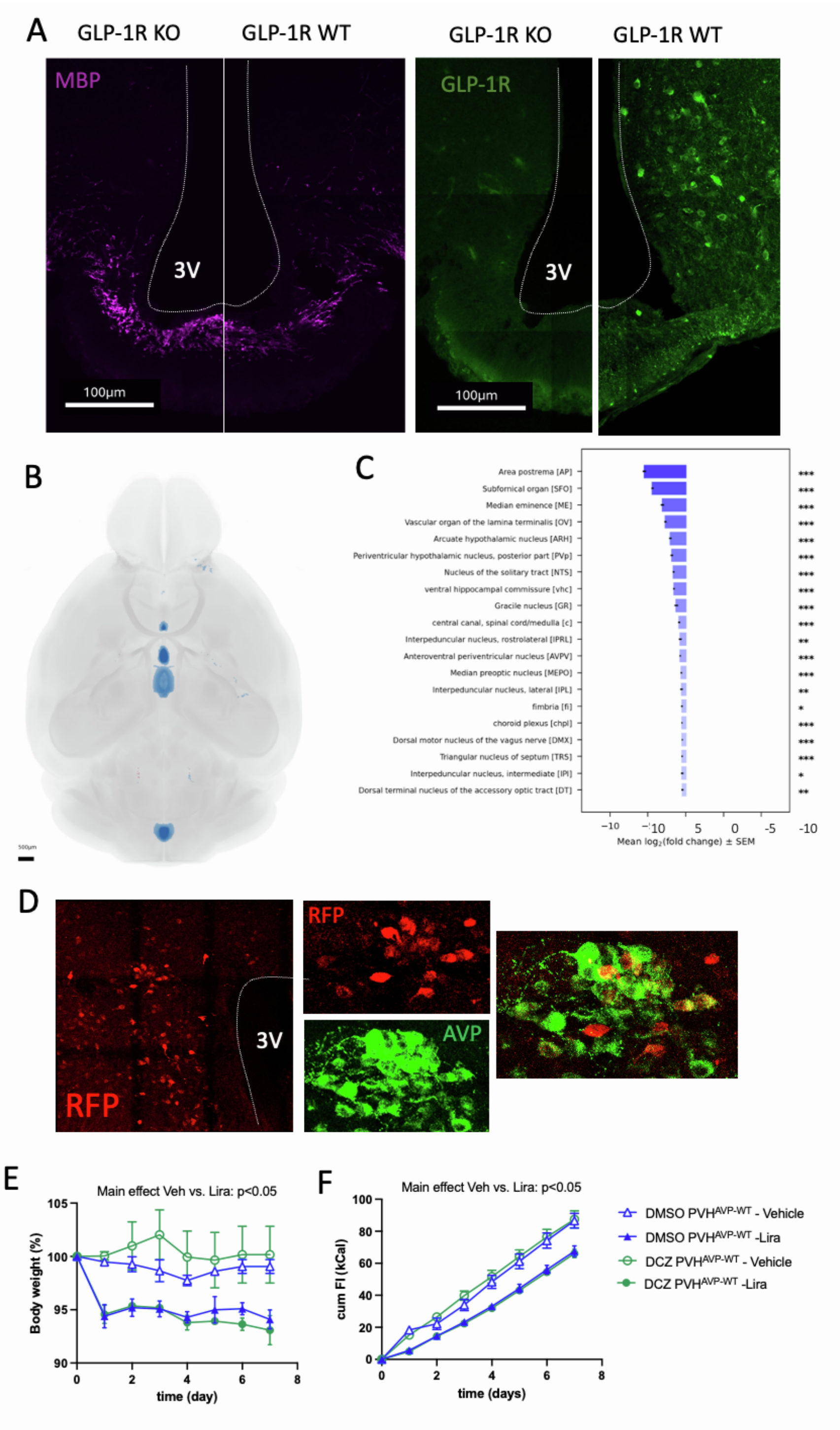

Supplementary video 1:

Representative movie demonstrating SAGLP-1RA^{IR800} distribution in DIO mouse brain 30 min after acute intravenous dosing. Fluorescence signal is shown in glow scale and brain autofluorescence in grey scale.

Supplementary video 2:
Representative movie demonstrating SAGLP-1RA^{IR800} distribution around the mediobasal hypothalamus (MBH) in DIO mouse brain 30 min after acute intravenous dosing. Fluorescent signal is shown in glow scale. The brain background autofluorescence is grey.

Supplementary Table 1

Top genes enriched in *Gipr*$^{+}$ oligodendrocytes vs. Gipr^{-} oligodendrocytes from the adult mouse median eminence.

Supplementary Table 2

Top pathways enriched in *Gipr*$^{+}$ oligodendrocytes vs. Gipr^{-} oligodendrocytes from the adult mouse median eminence.

Supplementary Table 3

Metabolites enrichment, in the eWAT, iBAT, Muscle, Plasma and liver of OL$^{GIPR-/-}$ vs OL$^{GIPR+/+}$ mice.
Metabolic pathway enrichment in the eWAT, Plasma, Liver and eWAT of OL$^{GIPR-/-}$ vs OL$^{GIPR+/+}$ mice.
Differential correlation analysis in the eWAT, Plasma and Liver of OL$^{GIPR-/-}$ vs OL$^{GIPR+/+}$ mice.
